# Sesterterpene ophiobolin biosynthesis involving multiple gene clusters in *Aspergillus ustus*

**DOI:** 10.1038/srep27181

**Published:** 2016-06-07

**Authors:** Hangzhen Chai, Ru Yin, Yongfeng Liu, Huiying Meng, Xianqiang Zhou, Guolin Zhou, Xupeng Bi, Xue Yang, Tonghan Zhu, Weiming Zhu, Zixin Deng, Kui Hong

**Affiliations:** 1 Key Laboratory of Combinatorial Biosynthesis and Drug Discovery (Wuhan University), Ministry of Education, and Wuhan University School of Pharmaceutical Sciences, Wuhan, 430071, PR China; 2 BGI-Shenzhen, Shenzhen 518083, China; 3 Key Laboratory of Marine Drugs, Ministry of Education of China, School of Medicine and Pharmacy, Ocean University of China, Qingdao 266003, China

## Abstract

Terpenoids are the most diverse and abundant natural products among which sesterterpenes account for less than 2%, with very few reports on their biosynthesis. Ophiobolins are tricyclic 5–8–5 ring sesterterpenes with potential pharmaceutical application. *Aspergillus ustus* 094102 from mangrove rizhosphere produces ophiobolin and other terpenes. We obtained five gene cluster knockout mutants, with altered ophiobolin yield using genome sequencing and *in silico* analysis, combined with *in vivo* genetic manipulation. Involvement of the five gene clusters in ophiobolin synthesis was confirmed by investigation of the five key terpene synthesis relevant enzymes in each gene cluster, either by gene deletion and complementation or *in vitro* verification of protein function. The results demonstrate that ophiobolin skeleton biosynthesis involves five gene clusters, which are responsible for C15, C20, C25, and C30 terpenoid biosynthesis.

Terpenoids are the most structurally and stereochemically diverse family of natural products, with more than 65,000 compounds identified^1^. Terpenoids are synthesized by plants, microorganisms and animals using simple five-carbon isoprene units of dimethylallyl diphosphate (DMAPP) and isopentenyl diphosphate (IPP) as building blocks. Prenyltransferases (PTs) catalyze chain elongation from these building blocks to yield linear polyisoprenoid diphosphates such as geranyl diphosphate (GPP, C10), farnesyl diphosphate (FPP, C15), geranylgeranyl diphosphate (GGPP, C20), geranylfarnesyl diphosphate (GFPP, C25) and hexaprenyl diphosphate (HexPP, C30). The polyisoprenoid diphosphates GPP, FPP, GGPP, GFPP and HexPP are then cyclized or rearranged by terpenoid synthase (TS) (or terpene cyclase (TC) for cyclized terpenes) to yield monoterpenes, sesquiterpenes, diterpenes, sesterterpenes and triterpenes, respectively^2^,^3^. Further biosynthetic modifications of these compounds are catalyzed by various enzymes including cytochrome P450 monooxygenases, oxidoreductases, hydrolases and different group transferases^2^. In filamentous fungi, genes coding for these enzymes together with those coding for specific regulatory function and resistance proteins are usually contiguously aligned in the genome^2^,^4^,^5^. Terpenoid diversity is facilitated by TS (or TC), the catalysis mechanisms of which are so complex that thus far our understanding thereof is still incomplete^6^.

Among the diverse and abundant terpenoids identified to date, sesterterpenes make up less than 2% (965 compounds) of all terpenoids^7^. Sesterterpenes are rare among marine fungi^8^ with many isolated from mangroves, which include two groups of neomangicols A–C and mangicols A–G sesterterpenes from the Bahamas mangrove *Fusarium* sp^9^,^10^; asperterpenoid A from a south China mangrove endophytic fungus *Aspergillus* sp. (an inhibitor of *Mycobacterium tuberculosis* protein tyrosine phosphatase B)^11^; and ophiobolins produced by *Aspergillus ustus* from south China mangrove rhizosphere^12^.

The FPP-derived artemisinin and GGPP-derived paclitaxel are well known antimalarial and anticancer drugs, respectively. Although there are currently no GFPP-derived drugs, ophiobolins which are unique sesterterpenes characterized by the tricyclic 5–8–5 ring system (Fig. 1, Supplementary Fig. S1), presented potential pharmaceutical applications. These compounds are assigned to subgroups A–T according to the order of discovery (Supplementary Fig. S1). Five genera of terrestrial and marine fungi including *Bipolaris, Cephalosporium, Aspergillus, Emericella* and *Ulocladium*^12^,^13^,^14^,^15^,^16^ reportedly produce these compounds. They were first discovered as fungal pathogen phytotoxins since they cause brown spot lesions on the leaves of rice, maize and sorghum. The pathogenic fungi formerly described as *Cochliobolus miyabeanus, Drechslera oryzae, Helminthosporium oryzae* and *Ophiobolus miyabeanus* have since been reclassified under the genus *Bipolaris*^15^. The term ophiobolin was adopted when this compound was isolated from the genus *Ophiobolus*^17^. Ophiobolins have attracted widespread attention due to their phytotoxic, antimicrobial, nematocidal and cytotoxic bioactivities^15^. Ophiobolin A, the first ophiobolin to be discovered was found to interact with calmodulin^18^, making it a useful calmodulin probe for research purposes^19^, and allowing its therapeutic application in anti-cancer^20^. It was recently reported that ophiobolin A has therapeutic potential for using as an anti-glioma agent^21^, as well as in the treatment of Parkinson’s disease^22^. Ophiobolin O holds promise in reversing adriamycin resistance in cancer cells^23^.

Despite their potential therapeutic value, our knowledge regarding ophiobolin biosynthesis is limited. In the 1960s, it was determined that the 5–8–5 ring system skeleton of ophiobolin was produced by cyclization of GFPP^15^. Though the discovery of sesterterpenes filled the gap between diterpenes (C20) and triterpenes (C30), little is known regarding the mechanism of sesterterpene synthease compared with that of sesquiterpene synthase, diterpene synthase and triterpene synthase^2^,^3^,^6^. To our knowledge, no sesterterpene biosynthetic pathway has been identified.

*Aspergillus ustus* 094102 was found to produce ophiobolin and four other terpenes, including the sesquiterpenoids drimane, isochromane, sterols and dipeptides^12^,^24^ (Fig. 1, Supplementary Fig. S1). To investigate ophiobolin biosynthesis mechanism, we carried genome sequencing and *in silico* analysis, combined with *in vivo* gene (cluster) deletion and complementation, and *in vitro* protein function verification processes. We found that the formation of ophiobolin skeleton in *A. ustus* involved five gene clusters related to C15, C20, C25 and C30 terpenoid biosynthesis.

## Results

### Genome sequencing and *in silico* analysis

At the time of our investigation (in May, 2011), the only information known about ophiobolin biosynthesis was that the 5–8–5 ring system skeleton is formed by cyclization of GFPP^15^, which is similar to mangicol biosynthesis^10^. Given the limited knowledge regarding sesterterpene biosynthesis, whole genome sequencing offered the best avenue for investigating this biosynthetic pathway. We completed genome sequencing and gene function annotation for *A. ustus* 094102 in April, 2012. The genome size of strain 094102 is 40.09 Mb, assembled as 301 contigs and 174 scaffolds, which included 13,982 protein-coding genes predicted by BlastNP searches against the SwissProt, GO, COG, KEGG and NR databases (Supplementary Table S1). Using this local database of the assembled genome, we scanned all TS (or TC) proteins manually and found 27 putative enzymes, which were classified to four groups including sesquiterpene, diterpene, and triterpene synthases and a ‘function unknown’ group of hypothetical proteins (Supplementary Table S2). The ‘function unknown’ group was further investigated since no sesterterpene synthase enzymes had been reported at that time and we speculated that this enzyme would likely be present in this group. Because chain length is the main feature differentiating sesterterpenes from other terpenoids and ophiobolin is a trans-IPP terpenoid, we performed chain length determination (CLD) analysis^25^ for the 15 predicted trans-IPP terpene synthesis proteins. Protein Au3446 in the ‘unknown function’ group was selected as probably containing the GFPP synthase (GFPPS) domain (Supplementary Fig. S2).

### *Au*3446 gene inactivation and complementation, and disruption of the POC3446 gene cluster

We first deleted the *Au*3446 gene in *A. ustus* 094102 by gene replacement, a modified version of a previously reported method^26^ using a deletion cassette as shown in Supplementary Fig. S3, the primers and plasmids are specified in Supplementary Tables S3 and S4, respectively. Compared with wild type *A. ustus* 094102, ophiobolin production was decreased (but not completely abolished) in the Au3446 mutant (∆*Au*3446) (Fig. 2). To confirm the role of the *Au*3446 gene in ophiobolin production, we complemented *Au*3446 back into the mutant strain ∆*Au*3446; ophiobolin yield was recovered in this complementary mutant strain (∆*Au*3446::*Au*3446, Fig. 2). This indicated that *Au*3446 was involved in the biosynthesis of ophiobolin but that it was likely not the key enzyme.

Considering that *Au*3446 might not be the only gene involved in ophiobolin synthesis in the gene cluster, and that the genes such as regulators among the cluster likely work together with *Au*3446, which would explain the incomplete abolishment of ophiobolin production following *Au*3446 deletion, we performed *in silico* analysis of the regions flanking *Au*3446 based on the structure of ophiobolins (Supplementary Fig. S1). This allowed us to construct a putative gene cluster (Fig. 3a), named POC (Presumed Ophiobolin biosynthesis gene Cluster) 3446. A large fragment knockout (10.706 kb) of this gene cluster was constructed using a method similar to that for single gene deletion. The deletion cassette was constructed using primers and plasmids specified in Supplementary Tables S5 and S6, respectively. However, the resulting mutant, ∆POC3446, also only partially abolished ophiobolin production (Fig. 3b), which implies that POC3446 was not the only gene cluster responsible for ophiobolin biosynthesis.

### Disruption of the other 14 gene clusters containing trans-IPP domain-coding genes

In order to find the gene cluster directly responsible for the production of ophiobolin, we further investigated the other 14 gene clusters that contain trans-IPP domain coding genes (including the diterpene and ‘function unknown’ group in Supplementary Table S2). Using the genetic manipulation system established to delete POC3446, we constructed deletion DNA cassettes for each gene cluster using the relevant primers and plasmids (Supplementary Tables S5 and S6), to obtain 14 gene cluster disruption mutants. Surprisingly, compared with the wild-type strain, deletion mutants of four other gene clusters (POC8003, POC6298, POC13192 and POC11565, (Fig. 3a)), had altered yields of ophiobolin and drimane (Fig. 3b). Production of ophiobolin was completely abolished in the ∆POC8003 mutant; increased significantly in the ∆POC6298 mutant (p < 0.05); was partially but significantly decreased in the ∆POC11565 (p < 0.05), ∆POC13192 (p < 0.05) and ∆POC3446 (p < 0.01) mutants, relative to the wild-type strain. At the same time, the yield of drimane was significantly increased in mutants ∆POC11565 (p < 0.05), ∆POC13192 (p < 0.05), ∆POC3446 (p < 0.01) and ∆POC8003 (p < 0.05), but significantly decreased in the ∆POC6298 (p < 0.01) mutants, relative to the wild-type strain (Fig. 3b, Supplementary Fig. S4). Since these mutants represent gene cluster deletions as opposed to single gene deletions from a single cluster, this suggests that multiple gene clusters (five in this study) are involved in ophiobolin and drimane biosynthesis. The five terpene synthesis relevant enzymes in these clusters (Fig. 3a) most likely have important roles in the network of ophiobolin and drimane biosynthesis.

### *In silica* analysis and functional prediction of the five TS proteins

Based on the updated terpene synthases sequences in the GenBank database available in 2013, phylogenetic analysis was performed using the amino acid sequences of the five proteins and their corresponding homologous proteins extracted from the database. The neighbor-joining phylogenetic tree showed that the five proteins were distributed in three clusters that correspond to three different enzyme types: di(sester) terpene synthase, hexprenyl pyrophosphate synthases (HexPPS), and **f**arnesyl pyrophosphate synthases (FPPS), which demonstrates the functional diversity of these enzymes (Fig. 4). Au8003, which was previously thought to be a diterpene synthase (Supplementary Table S2), clustered closely with AcOS, an ophiobolin F biosynthase discovered during a diterpene synthase screen in 2013^27^, and loosely with three other diterpene synthases: PaPs (*Phomopsis amygdali* phomopsene synthase)^28^, PaFS (*Phomopsis amygdali* fusicoccadiene synthase)^29^ and AbFS (*Alternaria brassicicola* fusicoccadiene synthase)^30^ (Fig. 4). BlastNP analysis also indicated that Au8003 shared a sequence identity of 65% with AcOS (Supplementary Table S7).

Meanwhile, Au13192 and Au11565 clustered with the same di(sester)terpene synthase branch but not as closely as Au8003 (Fig. 4); their amino acid sequences had the highest identities with PaFs at 36% and AcOs at 32%, respectively (Supplementary Table S7). Intriguingly these four enzymes (AcOS, PaFs, PaPs and AbFS) are all bifunctional terpene synthases that each possess two catalytically independent domains namely PT and TC, and can therefore carry out both chain elongation and terpene cyclization. A multiple sequence alignment using BIOEDIT software also showed that Au8003, Au13192, and Au11565 each contained both TC and PT domains (Supplementary Fig. S5). We therefore assumed that proteins Au8003 and Au11565 were sesterterpene synthase, and that Au13192 was diterpene synthase. Subsequent analysis (with sequences first made available in 2015) demonstrated more clearly the relationship of these three *A. ustus* proteins with the identified enzymes that coincide with these predictions (Supplementary Table S7 and Fig. S6).

The majority of matched sequences for Au6298 and Au3446 were hypothetical or predicted fungal proteins; the closest matching identified protein to Au6298 was a plant EpFPPS (*Euphorbia pekinensis* farnesyl diphosphate synthase), at an identity of 49%, and to Au3446 was a bacterial MlHexPPS (*Micrococcus luteus* heterodimeric hexaprenyl diphosphate synthase), at an identity of 32% (Supplementary Table S7). Au6298 and Au3446 were only loosely clustered with their neighboring sequences (Fig. 4). We therefore proposed that Au6298 may be an FPPS and Au3446 an HexPPS.

**Figure 1:**
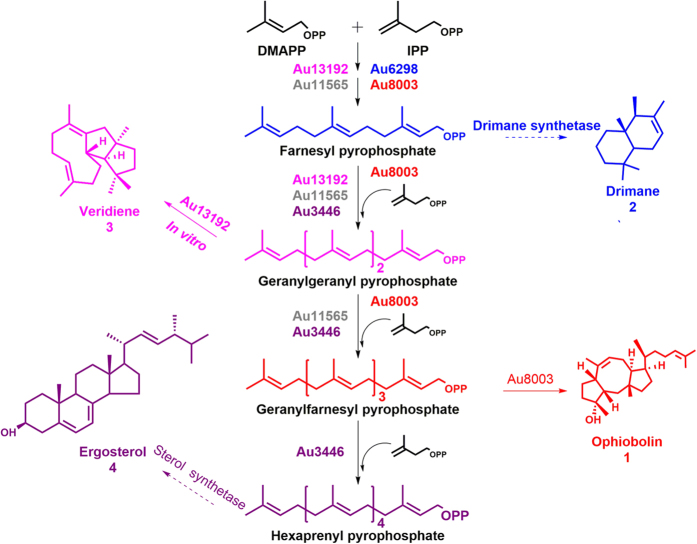
Hypothesized pathway of sesterterpene ophiobolin biosynthesis in *A. ustus.* Au8003 is the enzyme responsible for chain elongation from DMAPP and IPP to GFPP, as well as cyclization from GFPP to the end product ophiobolin (**1**). Production of ophiobolin is also connected with four other pathways via intermediates of DMAPP, IPP, FPP, GGPP, and GFPP. The enzymes Au6298, Au13192 and Au11565 could catalyze chain length elongation from DMAPP and IPP to the end products FPP, GGPP and GFPP, respectively. FPP could be used for drimane (**2**) synthesis by drimane synthetase or for HexPP synthesis by Au3446, which may then be used to synthesize ergosterol (**4**). GGPP produced by Au13192 (with the silent TC domain) is an important precursor for ophiobolin (**1**) production, but it can also produce veridiene **(3)** *in vitro*. GGPP or GFPP produced by Au11565 and Au3446 are also substrates for ophiobolin production.

**Figure 2:**
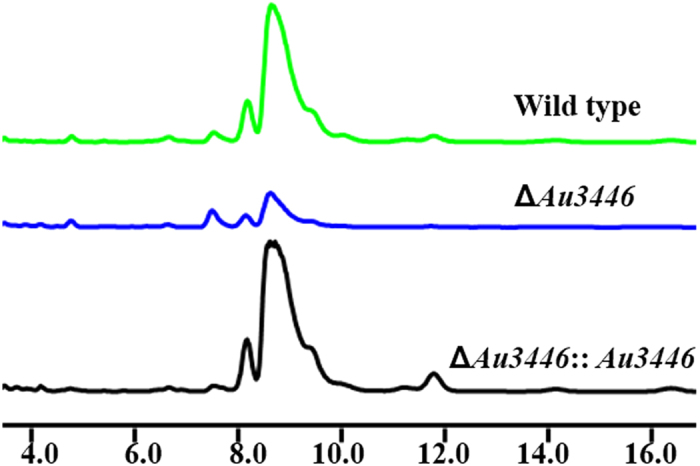
Au3446 gene deletion and complementation. HPLC analysis of ophiobolin production by wild type, Δ*Au*3446 mutant and the *Au*3446Δ::*Au*3446 complementary *A. ustus* strain of 094102.

Based on the functional prediction and gene cluster inactivation results, we hypothesized that POC8003 was responsible for the biosynthesis of ophiobolin, while the other four gene clusters were partially involved (Fig. 1).

### *In vitro* verification of Au8003, Au13192 and Au6298 enzyme activity

As the involvement in ophiobolin biosynthesis of Au3446 was confirmed by gene deletion and complementation, we didn’t test it further *in vitro*. For Au11565, we failed to obtain its active protein. The functions of the other three enzymes namely Au8003, Au13192 and Au6298 were examined *in vitro* as following. The Au8003 gene was amplified by RT-PCR and inserted into pET28a plasmid, and expressed as an *N*-terminal His_6_-tagged protein in *E. coli* BL21 (DE3) (Supplementary Fig. S7). The *in vitro* functional assay of the Au8003 fusion protein was performed using four substrates–DMAPP, GPP, FPP and GGPP, added IPP in the presence of Mg^2+^. GC-MS analysis showed that a peak appeared from reaction products of all four substrates at a retention time of 17 min (Fig. 5a). The corresponding molecular ion peaks were 55, 69, 81, 95, 107, 121, 135, 147, 161, 173, 187, 205, 229, 247, 325, 340, and 358, etc. (Fig. 5b), which were similar to that reported for ophiobolin F^27^. These results indicated that Au8003 can indeed catalyze chain length elongation via its PT domain, using DMAPP, GPP, FPP or GGPP as substrates to produce GFPP; which was then cyclized to the ophiobolin skeleton via its TC domain.

We amplified the Au13192 coding gene, which contained nine exons, by overlap extension PCR (Supplementary Fig. S8, Table S8) using *A. ustus* 094102 genomic DNA as template. The resulting gene was cloned and overexpressed using the same method as Au8003; the product was then purified as a His-tag-fusion protein (Supplementary Fig. S9). The protein was added to three reaction systems containing the substrates DMAPP, GPP or FPP, together with IPP, in the presence of MgCl_2_. The resulting solution was extracted with n-pentane, evaporated, and subjected to GC-MS analysis. The mass proton peaks (Fig. 5c,d) matched the newly discovered diterpene variediene^31^, which indicated that Au13192 has a similar function to EvVS (the diterpene synthase from the fungus *Emericella variecolor* with both PT and TC domains)^31^. With its two functional domains, Au13192 could potentially catalyze chain elongation of DMAPP, GPP and FPP to GGPP, and cyclize GGPP to variediene. However, no variediene-like compound was however detected among the *A. ustus* 094102 products, and the TC domain of Au13192 was therefore assumed to be inactivated under the culture conditions for ophiobolin synthesis.

The Au6298 fusion protein was obtained using a similar method as Au8003 (Supplementary Fig. S10). The *in vitro* test was performed with purified His-T7-tag- Au6298 recombinant protein using the substrates DMAPP and IPP, in the presence of Mg^2+^. The reaction product was treated with phosphatase to hydrolyze the diphosphate using the authentic FPP as control and the resultant products were detected by GC-MS. Au6298 was confirmed to catalyze conversion of DMAPP and IPP to FPP from the mass spectra of its *in vitro* reaction product hydrolysate (Fig. 5e,f), which matched that of authentic farnesol (Supplementary Fig. S11).

## Discussion

Although global regulatory networks and crosstalk between pathways are familiar concepts to biochemists, a network of pathways involving multiple gene clusters has not previously been reported for terpene biosynthesis to our knowledge. Based on the limited number of studies describing sesterterpene biosynthesis^10^,^15^, we inactivated gene clusters containing trans-IPP coding genes one-by-one, which allowed us to investigate each of their contributions to ophiobolin biosynthesis in *A.ustus* 094102. Using robust and comprehensive *in silico*, genetic and *in vitro* investigation techniques, we identified five gene clusters involved in ophiobolin synthesis, which was confirmed by examination of five key terpene synthesis relevant enzymes from each gene cluster: three proteins namely Au8003, Au13192 and Au6298, were shown to catalyze the synthesis of sesterterpene ophiobolin, diterpene veridiene and farnesyl diphosphate (FPP), respectively by *in vitro* function verification; Au3446 function was confirmed by deletion and complementation mutants, and it was predicted as an HexPPS (*Aspergillus ustus* hexaprenyl diphosphate synthase) by *in silico* analysis; the fifth protein Au11565 was predicted as another sesterterpene biosynthesis enzyme (GFPPS/TC).

A similar pathway network mechanism was recently reported in *Artemisia annua* where manipulating the amorpha-4,11-diene synthase gene not only affected artemisinin synthesis but also changed the non-amorphadiene sesquiterpene and genome-wide volatile profile^32^, which suggests the network-dependent biosynthesis of artemisinin. On the contrary, a trifunctional (all-E)-GFPP/HexPP/HepPP (heptaprenyl diphosphate) synthase from *Bacillus clausii*, catalyzed intermediates of two isopronoid pathways^33^, which may represent another kind of pathway network based on the multi-functional role of GFPPS. Similarly, the diterpene synthase EvVS from the fungus *Emericella variecolor* is a chimeric terpene synthase with both TC and PT domains, which enables production of both di- and sesterterpenes^31^. Au8003 and Au13192 do not appear to have these dual capabilities as we did not find other C20 or C25 terpenoids, respectively among their *in vitro* reaction products; this indicates that ophiobolin and drimane are not synthesized by this type of network in *A. ustus* 094102.

C25 sesterterpene synthase investigation represents only a small fraction of the vast terpenome. Following clarification of the cyclization mechanism of the 5–8–5 ring skeleton from GFPP^15^, ophiobolin F synthase (AcOS) was only discovered by accident in more than 40 years later, during genome mining for diterpene synthase from *Aspergillus clavatus*^27^. AcOS was the first reported sesterterpene synthase and it shares homology with fusicoccadiene synthase (PaFS)—an unusual bifunctional class-I diterpene synthase that has two catalytically independent domains: prenyltransferase and terpene cyclase^29^. Two other sesterterpene synthases were recently identified: NfSS for sesterfisherol biosynthesis from *Neosartorya fischeri*^34^, and EvSS for stellatic acid biosynthesis from *Emericella variecolor*^35^. EvVS for variediene (C20) biosynthesis also showed potential for C25 terpenoid synthesis^31^. NfSS, EvSS and EvVs were all discovered using the C-terminal PT and N-terminal TC domains as query probes for genome mining, which are unique features of sesterterpene synthases.

Au8003 was grouped with its closest neighbor AcOS, which was discovered in 2013, in the di(sester)terpene synthases cluster (Fig. 4). After EvSS^35^ was added in 2015, a sesterterpene synthase clade (A1) was clearly separate from the other diterpene synthase clade (A2) (Fig. S6). Similarly, Au11565, a presumed terpene synthase (Table S2), was grouped with NfSS^34^, a sesterterpene synthase at clade B1 (Fig. S6). Au13192, which was confirmed to be a diterpene synthase, clustered closely with the diterpene synthases EvVS^31^, in clade B2 (Fig. S6). This indicates that the amino acid sequence clustering not only reflects the terpene cyclization mechanism^31^,^34^, but also correlates with the product chain length. Though we could not identify the function of Au11565 *in vitro* at present, this analysis revealed that it could be another sesterterpene synthase so we are currently investigating.

**Figure 3:**
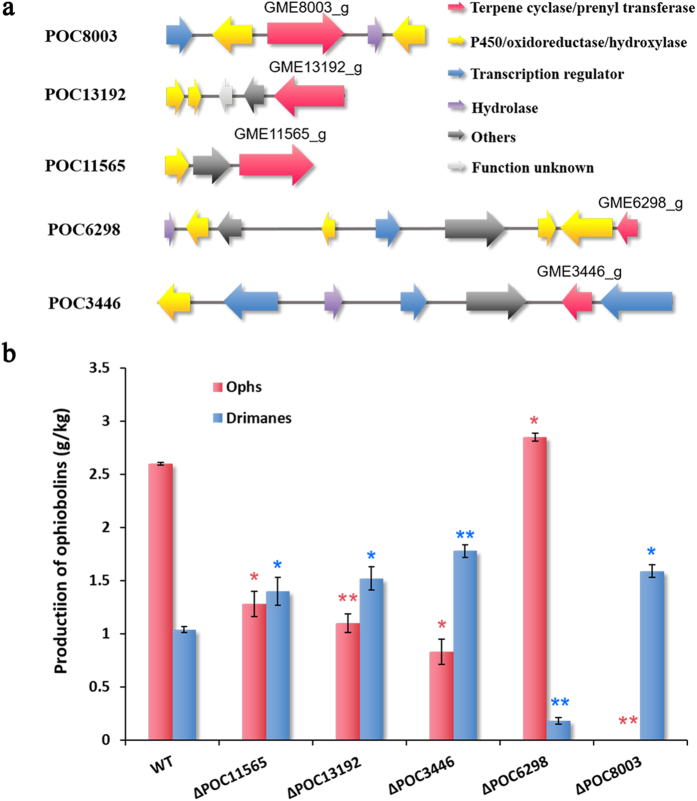
Gene cluster inactivation. **(a)** Five gene clusters of *Aspergillus ustus* are involved in ophiobolin synthesis. Red: terpene cyclase(or prenyl transferase); yellow, cytochrome P450 or oxidoreductase or hydroxylase; blue, transcription regulator; purple, hydrolase; black, other function; gray, function unknown. Numbers on or near the open reading frame are gene codes. (**b**) Production of ophiobolin and drimane by the five mutants relative to the wild type strain analysed by HPLC. Data are means ± SE of at least five independent measurements. *P < 0.05, **P < 0.01 *vs* wild type strain, student’s t-test.

**Figure 4:**
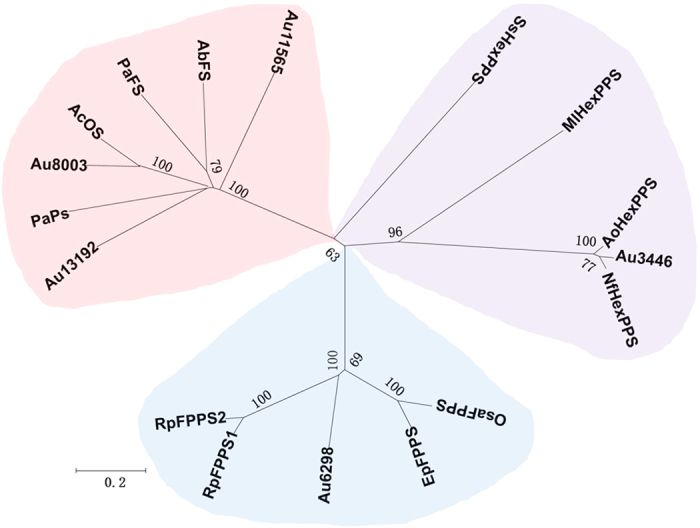
Neighbour-joining phylogenetic tree based on amino acid sequences of bifunctional diterpene and sesterterpene synthases, farnesyl pyrophosphate synthases and hexaprenyl pyrophosphate synthases. The terpene synthases involved in ophiobolin biosynthesis are found in three clusters. light orange: bifunctional di(sester) terpene synthases; light purple: hexaprenyl pyrophosphate synthases; light blue: farnesyl pyrophosphate synthases. The tree was generated based on the Kimura 2-parameter matrix in MEGA software (version 5.0). Numbers at nodes are bootstrap values obtained using bootstrapping with 1000 repetitions. The scale bar corresponds to 0.2 substitutions per nucleotide position. *Aspergillus clavatus* ophiobolin F synthase (AcOS A1C8C3), *Phomopsis amygdali* phomopsene synthase (PaPS AB254159), *Phomopsis amygdali* fusicoccadiene synthase (PaFS AB267396), *Alternaria brassicicola* fusicoccadiene synthase (AbFS C9K2Q3), *Ornithogalum saundersiae* farnesyl pyrophosphate synthase (OsaFPPS KF509889), *Rhopalosiphum padi* isoprenyl diphosphate synthase (RpFPPS1 HQ850372 and RpFPPS2, HQ850373), *Euphorbia pekinensis* farnesyl diphosphate synthase (EpFPPS FJ755465), *Sulfolobus Solfataricus* Hexaprenyl Pyrophosphate Synthase (SsHexPPS 2AZK_B), *Micrococcus luteus* heterodimeric hexaprenyl diphosphate synthase (MlHexPPS 3AQB_B), *Aspergillus oryzae* hexaprenyl pyrophosphate synthase (AoHexPPSXP_001824458), *Neosartorya fischeri* hexaprenyl pyrophosphate synthetase (NfHexPPS XP_001263839).

**Figure 5:**
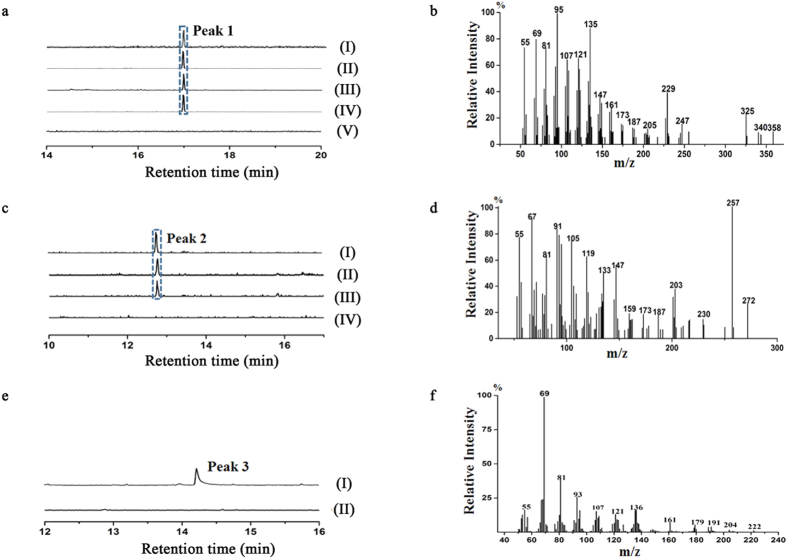
GC-MS profiles of the products of *in vitro* enzymatic reactions. The chromatograms of Au8003 reaction product ophiobolin F extracted at m/z 358 (**a**): (I) GGPP+IPP, (II) FPP+IPP, (III) GPP+IPP, (IV) DMAPP+IPP and (V) IPP, and MS Spectra of peak 1 (**b**); chromatograms of Au13192 reaction product veridiene extracted at m/z 272 (**c**): (I) FPP+IPP, (II) GPP+IPP, (III) DMAPP+IPP, (IV) IPP and MS Spectra of peak 2 (**d**); chromatograms of phosphatase hydolysate of Au6298 reaction product FPP extracted at m/z 222 (**e**): (I) DMAPP+IPP, (II) IPP only and MS Spectra of peak 3 (**f**).

Based on the function verification of the five key enzymes related to ophiobolin synthesis in *A. ustus*, the results from gene cluster deletion (Fig. 3) can be interpreted as follows: The significant decrease in ophiobolin production (Fig. 3b) for ∆POC13192, ∆POC11565, and ∆POC3446 suggested that these three gene clusters were involved in the synthesis of precursor compounds for ophiobolin biosynthesis. Inactivation of POC6298 lead to an increase in ophiobolin (p < 0.05) and a decrease in drimane (p < 0.01) (Fig. 3b), which indicates the importance of the POC6298 gene cluster in drimane biosynthesis as well as the competition for precursors between these enzymes and POC8003, which may well represent the primary pathway for ophiobolin synthesis. Since FPP is one of these sesterterpene precursors, deletion of the gene cluster including the Au6298 gene was expected to decrease the yield of ophiobolin if Au8003 uses FPP as substrate. The increase in ophiobolin may indicate that DMAPP and IPP are key precursors used simultaneously for drimane biosynthesis by Au6298 and for ophiobolin synthesis by Au8003 and, in the absence of Au6298, more substrate is available to Au8003. Further investigation of drimane cyclase in *A. ustus* 094102 is needed to clarify the relationship between these two gene clusters. Since deletion of POC3446 significantly increased drimane production (Fig. 3b), FPP could be a substrate for Au3446; and the decrease in ophiobolin for ∆POC3446 showed that Au3446 could produce substrate for Au8003—it therefore increased production of longer linear polyisoprenoid diphosphates using FPP and IPP as substrates to produce GGPP, GFPP, and HexPP, similar to the identified HexPPS from *Micrococcus luteus*^36^. In *Arabidopsis thaliana* four GFPPS and one PPPS (C ≥ 30, polyprenyl pyrophosphate synthase) were identified from ten formerly presumed GGPPSs^37^, which demonstrates the close relationship between GGPPS, GFPPS and PPPS.

In conclusion, we provide a theoretical biosynthesis pathway for ophiobolin (**1**) production in *A. ustus*, which also involves pathways for the production of drimane (**2),** veridiene (**3)** and ergosterol (**4)** (Fig. 1). Thus, Au8003 in POC8003 is responsible for chain elongation from DMAPP and IPP to GFPP, as well as cyclization of GFPP to the end product ophiobolin (**1)**. Although POC8003 is the primary gene cluster responsible for ophiobolin biosynthesis, four other pathways (highlighted by the four terpene synthase relevant proteins) are involved via the intermediates of DMAPP, IPP, FPP, GGPP and GFPP. Enzymes Au6298, Au13192 and Au11565 could catalyze chain length elongation from DMAPP and IPP to produce FPP, GGPP and GFPP, respectively. The POC8003 and POC6298 pathways appear to compete for DMAPP and IPP, and FPP is not consumed directly by Au8003, but could be used for drimane (**2**) synthesis by drimane synthetase, or for HexPP synthesis by Au3446, which may then be converted to sterol ergosterol (**4)**. GGPP, synthesized by Au13192, which has a silent TC domain, was an important precursor for ophiobolin production. GGPP or GFPP synthesized by Au11565 and Au3446, respectively are also substrates for ophiobolin production.

Terpenoids are not simply secondary metabolites since some terpenes, including respiratory quinones, the cell membrane component ergosterol in fungi and lipid diterpenes in archaea, are related to cell growth and development. The structural and functional diversity of terpenoids may necessitate a more complex biosynthetic pathway. It is plausible that interactions may occur between all possible pathways with overlapping precursors in terpenoid biosynthesis network. The complexity of terpene biosynthesis is also demonstrated by the recently reported involvement of multi-functional cytochrome P450 in the fumagillin pathway^38^ and the involvement of heteromeric GGPPS in monoterpene biosynthesis^39^. Because of the industrial and pharmaceutical importance of terpenoids, terpene compounds are an important target for microbial engineering^40^,^41^,^42^. Elucidation of this pathway network may therefore guide applications in metabolic engineering and synthetic biology. The whole genome sequences, bioinformatic tools, and bio-editing techniques and related experimental verification strategies presented here will be of great interest in further clarifying the complex, fascinating process of terpenoid biosynthesis.

## Methods

### Strains and media

*Aspergillus ustus* 094102 was isolated from a mangrove rhizosphere soil sample by our group in 2003 and deposited to the China Center for Type Culture Collection (CCTCC No. M 208153). Strain 094102 was cultivated on potato dextrose agar at 28 °C and stored at 4 °C. Fungal DNA and RNA from strain 094102 were extracted from liquid culture medium grown at 28 °C, 220 rpm for three days as previously described^24^. For the detection of ophiobolin and drimane, the wild type strain 094102 and its mutants were grown on solid medium containing corn niblet (100 g), yeast extract (1.7 g), ammonium tartrate (4.74 g), MgSO_4_ (0.85 g), KH_2_PO_4_ (0.5 g), sea salt (1.6 g) and tap water (40 g) at 20 °C for 18 days. *Escherichia coli* DH5α and TOP10 (Cwbio, Beijing) were used for gene manipulation using plasmids grown in Luria-Bertani (LB) medium and were selected with appropriate antibiotics. *E. coli* BL21 (DE3) (Novagen) was used to express *Au*8003, *Au*13192 and *Au*6298.

### General molecular biology experiments

PCR was performed using Taq DNA polymerase (Thermo Scientific) or NovoStar FastPfu (NovoGene, Beijing). PCR products were confirmed by DNA sequencing. DNA restriction enzymes were used as recommended by the manufacture (New England Biolabs). RNA extraction was performed using the RNeasy Mini Kit (Qiagen) and complementary DNA (cDNA) was reverse transcribed from total RNA using the Revert Aid First Strand cDNA synthesis Kit (Thermo Scientific). The Gel Extraction Kit (Biomiga, Beijing) was used for DNA purification; the Plasmid Miniprep Kit (MicLab Biotech) was used for plasmid extraction; and the pEasy-Blunt Cloning Kit (TransGen Biotech) was used for gene cloning.

### Chemicals

Isopentenyl pyrophosphate (IPP), dimethylallyl pyrophosphate (DMAPP), geranyl diphosphate (GDP), farnesyl diphosphate (FDP), and geranylgeranyl diphosphate (GGDP) were purchased from Sigma–Aldrich.

### HPLC analysis for ophiobolin and drimane production

HPLC analysis was performed using a Waters 2998 series system with Phenomenex Gemini column (C18 250 × 4.6 mm, 5 μm; Waters, Milford, MA, USA) at 25 °C. The Photo-Diode Array (PDA) detector was used to monitor ophiobolin at 234 nm and drimane at 381 nm. A full wavelength range of 210–400 nm was collected for complete analysis. Elution was carried out under isocratic conditions with the mobile phase consisting of methanol and water (85:15, v/v) for 30 min at a flow rate of 1.0 ml min^−1^, with an injection volume of 10 μl. Stock solution of ophiobolin G (>96% purity) was prepared in methanol at a target concentration of 10 gl^−1^. Peak areas at 234 nm were summed for ophiobolins and at 381 nm for drimanes.

### GC-MS analysis for *in vitro* protein products

Measurements were performed on a Varian 450 system (Varian, USA) hyphenated with a Varian triple quadrupole 320 mass spectrometer (Varian, Palo Alto, California, USA) with a Varian FactorFour VF-5 ms capillary column (30 m × 0.25 mm, 0.25 μm film thickness). For the *in vitro* reaction product (sesterterpene and diterpene) analysis, each sample was injected into the column at 60 °C in the splitless mode. After a 2 min isothermal hold at 60 °C, the column temperature was increased to 150 °C at 30 °C min^−1^, from 150–180 °C at 10 °C min^−1^, from 180–210 °C at 2 °C min^−1^ with a 5 min isothermal hold at 210 °C. The helium carrier gas flow rate was 0.66 ml min^−1^. For farnesol analysis, sample was injected into the column at 80 °C in the splitless mode. After a 2 min isothermal hold at 80 °C, the column temperature was increased to 290 °C at 10 °C min^−1^, with a 5 min isothermal hold at 290 °C. The helium carrier gas flow rate was 0.66 ml min^−1^.

### Genome sequencing

*Aspergillus* sp. 094102 genomic DNA was extracted via the cetyltrimethylammonium bromide (CTAB) method^43^ and submitted for whole genome sequencing by the Beijing Genomics Institution (BGI) in Shenzhen, China. Two types of 500 bp and 6 Kb sequence libraries were constructed using 5 μg and 20 μg DNA, respectively for which a total of 1.58 Gb and 1.16 Gb reads were generated by Illumina Hiseq™ 2000. Before assembling reads, reads with low quality bases (Phred quality score ≤ Q20), or ≥9 bp Ns (Ns = unknown bases), or ≥15 bp overlap between adapter and duplications sequences were filtered out. Short reads from the two libraries were assembled using SOAPdenovo 1.05^44^,^45^, with optimal assembly acquired using a key parameter (K) of 35.

### *In silico* analysis

Gene prediction was done by determining putative open reading frames with GeneMark-ES 2.3e^46^. Protein-encoding genes were annotated through BLASTP searches against the SwissProt (2012–04), GO (release:1.419), COG (release: 20090331), KEGG (release: 59), and NR (2012–04) databases, at a threshold e-value ≤ 1 × e-5. The best hit was filtered using a 50% identity threshold. Terpene synthesis genes were searched manually using “terpenoid synthase” or “terpenoid cyclase” as query words from the local genome database, and checked using BLASTX analysis in the GenBank database. Chain length determination site was analyzed as described by Hisashi *et al*.^25^. MEGA (version 5.0)^47^ was used for amino acid phylogenetic analysis. Multiple sequence alignments were performed using the BioEdit 7.0 software package.

### Generation of the *Au*3446 gene deletion cassette

The DNA cassette used for gene replacement was generated by applying the principle of a previously described method^26^. However instead of using fusion PCR, three fragments in the DNA cassette namely selective marker, right and left homologous sequences were fused via recombination to vector by the following steps: first, the *Streptoalloteichus hindustanus ble (Sh ble*) gene (1172 bp, together with the Ptef1 and Pem7 promoters for gene expression from *Saccharomyces cerevisiae* and *E. coli*, and the transcription termination region Tcyc1 from *S. cerevisiae*) was used as the selection marker, derived from pGAPZαA (Invitrogen) using the *Sh ble* primer pair (Supplementary Table S3); *Sh ble* was then transferred to the pMD18-T TA cloning vector to construct the plasmid pMD18-T-Sh ble (pWHU2201, Supplementary Table S4), which was verified by PCR and sequencing. Secondly, fragments *Au*3446L′ (1224 bp) and *Au*3446R′ (1657 bp) were amplified from *A. ustus* 094102 genomic DNA using primers *Au*3446L′-s/a, and *Au*3446R′-s/a, to construct the plasmids pMD18-T-Simple-3446L′ and pMD18-T-Simple-3446R′, which were verified by PCR and sequencing. pMD18-T-*Sh ble* and pMD18-T-Simple-3446L′ were digested using *Eco* RI and *Kpn* I, respectively; the resultant 3906 bp and 1218 bp fragments were ligated using T4 DNA ligase, and transformed into *E. coli* DH5α, to construct pMD18-T-Simple-3446L′-Sh ble, which was verified by *Eco* RI (5116 bp) single digestion and *Eco* RI and *Kpn* I double (1210 bp + 3906 bp) digestion. pMD18-T-3446L′-Sh ble and pMD18-T-Simple-3446R′ were digested with *Pst* I and *Hind* III, the resulting 5100 bp and 1642 bp fragments were ligated and transformed into *E. coli* DH5α to construct pMD18-T-3446L′-Sh ble-3446R′ (pWHU2203, Table S4), which was verified by *Hind* III single digestion (6750 bp) and *Pst* I and *Hind* III double digestion (1650 bp + 5100 bp). The cassette “3446L′-Sh ble-3446R′” was obtained by *Eco* RI and *Hind* III double digestion (4115 bp + 2635 bp) from the constructed plasmid (Supplementary Fig. S3a,b).

### *Au*3446 gene deletion

The protoplast of strain 094102 was obtained using media and an osmotic solution^48^ as following: glucose minimum media (GMM: glucose 5 g, 20× nitrate salts 25 ml, trace elements 500 μl, agar 20 g, in 500 ml dH_2_O, at pH 6.5) was used to obtain a large amount of fungal spores; GMM without agar was then used for spore germination. GMM with sorbitol 218.6 gl^−1^ (SMM) was used for protoplast regeneration and screening of transformants. The osmotic solution for protoplast preparation consisted of: MgSO_4_ (147.9 g) and 2 M NaPB (2.5 ml), in one liter dH_2_O; the pH was adjusted to 5.8 with 1 M Na_2_HPO_4_. Transformation was carried out after 10–11 hr of spore germination and treated with driselase for 3.5–4 hr. Transformants were selected on SMM with zeocin (1000 mgl^−1^) and checked by PCR using primers *Au3446*-s/a and *Sh ble*-s/a. *Sh ble-*positive but *Au3446*-negative transformants were selected and verified by PCR (Supplementary Fig. S3c).

### ∆*Au*3446 gene complementation

The hydromycin resistance gene *Hpt* II (2146 bp, together with the promoter pCaMV35S) was used as a selective marker for complementation. Plasmid pCAMBIA 1301 was linearized using *Sph* I and used as a template for PCR with primers *Hpt* II-s/a (Supplementary Table S3) to obtain the marker. Then *Hpt* II was cloned into pMD18-T to produce pUC18- *CaMV35S-HPH-CaMV35S polyA* (pWHU2202, Supplementary Table S4) and checked by sequencing. The upstream fragment of *Au*3446*-Au*3446L′ and downstream *Au*3446R′ were amplified from *A. ustus* 094102 genomic DNA using primers *Au*3446L′-s/a, and *Au*3446R′*up*-s/a, and verified by PCR and sequencing. The confirmed fragments were ligated using T4 DNA ligase to either side of the selective marker, to obtain the fragment*Au3446-Au3446L′-CaMV35S-HptII-CaMV35SpolyA-Au3446R′* (pWHU2205, Supplementary Table S4). This fragment was transformed into the protoplast of strain *A. ustus* 094102 ∆*Au3446* to obtain the complementary mutant by homologous recombination. Transformants were selected on SMM with zeocin (1000 mg l^−1^) and verified by PCR using primers *Hpt* II -s/a and *Sh ble*-s/a. Positive transformants were selected and verified by PCR.

### Gene cluster inactivation

Deletion cassettes for presumed gene clusters POC3446, POC6298, POC8003, POC11565 and POC13192 were generated using a similar method of gene deletion as described above. Briefly, approximately 1000–1500 bp of 5′ UTR and 3′ UTR were PCR-amplified from each target loci from *A. ustus* 094102 genomic DNA using the relevant primers (Supplementary Table S5) and ligated with the selective marker *Sh ble* and vector to obtain each plasmid (Supplementary Table S6). Transformants were obtained and selected as described in the previous section. For each of the five disruptions at least six transformants were picked and tested for ophiobolin and drimane production.

### Construction of plasmids for *Au*8003, *Au*6298, and *Au*13192 expression in *E. coli*

*Au*8003 and *Au*6298 cDNA were obtained by RT PCR from the genomic RNA of strain 094102 using primers in Supplementary Table S8. *Au*13192 cDNA was amplified by overlap extension PCR (Supplementary Fig. S8) using genomic DNA of *A. ustus* 094102 as template. Seven pairs of primes were designed as shown in Supplementary Table S8 for nine exons in *Au*13192, making the adjacent exon fragment overlap area about 20 bp. The PCR product of *Au*8003 was ligated to pEASY-Blunt to yield pEASY-Blunt-*Au*8003. After verification by sequencing, the *Hind* III-*Nde* I fragments from pEASY-Blunt vector was ligated to the same site of pET28a(+) to make pET28a(+)-*Au*8003. Similarly, pET28a(+)-*Au*6298 and pET28a(+)-*Au*13192 were obtained, with suitable restriction sites, as shown in Supplementary Table S8.

### Expression of *Au*8003, *Au*6298 and *Au*13192

*E. coli* BL21 (DE3) containing pET28a(+)-*Au*8003, pET28a(+)-*Au*6298 and pET28a(+)-*Au*13192 were applied to produce a His-T7-tag fusion protein of each gene product. The overnight seed cultures of recombinant bacteria were obtained on LB broth at 37 °C with 50 μgl^−1^ kanamycin. Expression of the fusion protein was induced by addition of 0.1 mM isopropyl β-D-thiogalactopyranoside (IPTG), when *E. coli* cells reached an absorbance of 0.6 at A_600_ and further incubated at 37 °C for 3–5 hr. To examine the expression of the recombinant genes, total cell extracts were obtained from 1 ml of the above culture by centrifugation and washed twice, disrupted by sonication and fractionated on 10% sodium dodecyl sulfate polyacrylamide gel electrophoresis (SDS-PAGE).

### Purification of Au8003, Au6298 and Au13192

Recombinant *E. coli* was cultured in 500 ml LB broth, after inducing with IPTG, and further incubated at 16 °C for 16–20 hr. Cells were harvested by centrifugation at 6000 rpm; resuspended in 20 mM HEPES[4-(2-hydroxyethyl)-1-piperazineethane-sulfonic acid], pH 8.0, 10% of glycerol, and 1 mM of DTT (dithiothreitol); and disrupted by sonication. After centrifugation, the supernatant was applied to a Ni-NTA column to purify His-T7-tag-Au8003, His-T7-tag-Au6298 and His-T7-tag-Au13192, respectively. Enzyme purity was determined by SDS-PAGE.

### *In vitro* assays of Au8003, Au13192 and Au6298 function

*In vitro* assays were carried out to verify Au8003, Au13192 and Au6298 function. To verify the function of Au8003 and Au13192, we used a modified version of the reaction system specified by Chiba *et al*.^27^ His-T7-tag-Au8003 recombinant protein (6.1 μM) was added into 200 μl reaction buffer containing 20 mM HEPES, 10% glycerol, 5 mM DTT, 5 mM MgCl_2_, 50 μM GGPP (or FPP, GPP, DMAPP) and 50 μM IPP, incubated at 30 °C for 3 h. The whole was extracted with ethyacetate (200 μl), which was then evaporated and a portion subjected to GC–MS analysis.

For *in vitro* assay of Au13192, His-T7-tag-Au13192 recombinant protein (6.2 μM) was added to 200 μl reaction buffer containing same components of those for Au8003 except using GGPP as substrate, incubated at 37 °C for 3 h. The resulting solution was extracted with n-pentane (200 μl) and evaporated, a portion was subjected to GC-MS analysis.

For Au6298, purified His-T7-tag- Au6298 recombinant protein (4.2 μM) was added to 600 μl reaction buffer containing 20 mM HEPES, 5 mM DTT, 5 mM MgCl_2 _and 50 μM DMAPP and 100 μM IPP, which was incubated at 30 °C for 5 h. The mixture was extracted with l-butanol saturated with water and then treated with potato acid phosphatase at 37 °C to hydrolyze the diphosphate moiety according to Fuji *et al*.^49^. The hydrolyzed products (FPP-derived farnesol) were extracted with hexane and analyzed by GC-MS.

## Additional Information

**How to cite this article**: Chai, H. *et al*. Sesterterpene ophiobolin biosynthesis involving multiple gene clusters in *Aspergillus ustus. Sci. Rep.* **6**, 27181; doi: 10.1038/srep27181 (2016).

## Supplementary Material

Supplementary Information
