# Supplementary material for: Sesterterpene ophiobolin biosynthesis involving multiple gene clusters in Aspergillus ustus
Source: Sci Rep. 2016 Jun 7;6:27181. doi: 10.1038/srep27181 (PMC4895135; doi:10.1038/srep27181)
Supplement: Supplementary Information [file srep27181-s1.pdf]

Supplementary Information

Sesterterpene ophiobolin biosynthesis involving multiple gene clusters in *Aspergillus ustus*

Hangzhen Chai[1], Ru Yin[1], Yongfeng Liu[2], Huiying Meng[1], Xianqiang Zhou[2], Guolin Zhou[1], Xupeng Bi[2], Xue Yang[1], Tonghan Zhu[3], Weiming Zhu[3], Zixin Deng[1], Kui Hong[1]

[1]Key Laboratory of Combinatorial Biosynthesis and Drug Discovery (Wuhan University), Ministry of Education, and Wuhan University School of Pharmaceutical Sciences, Wuhan, 430071, PR China

[2] BGI-Shenzhen, Shenzhen 518083, China

[3] Key Laboratory of Marine Drugs, Ministry of Education of China, School of Medicine and Pharmacy, Ocean University of China, Qingdao 266003, China

Corresponding to K.H. (email: kuihong31@whu.edu.cn)

Table S1. *Aspergillus ustus* 094102 genome assembly and main feature

**Statistic**	**Value**
Coverage	>68×
Genome assembly size (Mb)	40.09
Number of scaffolds	174
Scaffold N50(Kb)	1,814
Number of contigs	301
Contig N50(Kb)	675
CDS total length (Mb)	20.44 (50.98% of genome)
CDS average size (bp)	1461.62
Predicted protein-coding genes (Number)	13,982
Predicted protein-coding genes, total length (Mb)	23.19
Predicted protein-coding genes, mean length (kb)	1658.33
Exon total length (Mb)	20.44
Exon total number	43,400
Exon average length (bp)	470.88
Intron total length (Mb)	2.75
Intron total number	29,418
Intron average length (bp)	93.50
GC content (%)	51.22

Note: The gene density is 349 genes/ Mb scaffold. On average, the predicted genes contain 3.10 introns that are 471 bp long. This is in the range seen for other fungi, where the average intron densities range from just over 1.0 intron/ kb coding sequence (cds) in *Schizosaccharomyces pombe* to approximately 5.0 introns/ kb cds in *Cryptococcus neoformans*. For all of the predicted proteins, 8,882 had hits in the SwissProt database, 7,929 genes were mapped in the Kyoto Encyclopaedia of Genes and Genomes (KEGG) database, and 5,061 were classified in the Clusters of Orthologous Groups (COG) database, 8,336 were classified in the Gene Ontology (GO) database, 12,785 in the non-redundant database of NCBI (NR),and 10,928 in the Tremble database, 13,982 were hitted in all the above databases.

Table S2 Blastnp of the presumed 27 terpene synthases in *A. ustus* 094102 genome (done in Oct, 2012)

Presumed AUPTS type	AUPTS ID (Au)	AA No.	Intron No.	RTS	Max score	Query coverage	Max identity
**Sesqui AUPTS**	226	382	8	|EEA21257.1| pentalenene synthase, putative [*Penicillium marneffei* ATCC 18224]	125	95%	24%
	12847	408	8		116	83%	24%
	13884	363	8		141	93%	29%
	6852	390	8	|XP001390370.2| pentalenene synthase [*Aspergillus niger* CBS 513.88]	457	91%	62%
	11092	340	0	|ACZ56398.1| trichodiene synthase [*Fusarium incarnatum*]	126	69%	31%
**Di AUPTS**	6061	343	0	**|BAF45925.1| fusicoccadiene synthase [*Phomopsis amygdali*]**	207	97%	37%
	6241	744	7		288	89%	29%
	7240	762	8		258	94%	28%
	8003	725	3		517	100%	39%
	11189	710	5		292	94%	30%
	13192	708	8		425	98%	36%
	13606	755	7		307	85%	31%
	13676	717	9		294	85%	30%
**Tri AUPTS**	1332	430	2	|EIT75610.1| squalene synthetase [*Aspergillus oryzae* 3.042]	501	91%	59%
	7332	470	3		816	97%	83%
	1334	642	2	|XP001388569.2| squalene-hopene-cyclase [*Aspergillus niger* CBS 513.88]	655	95%	51%
	1561	382	1	|GAA92416.1| squalene/phytoene synthase [*Aspergillus kawachii* IFO 4308]	560	100%	71%
	5911	735	3	|EAW24229.1| oxidosqualene:lanosterol cyclase [*Neosartorya fischeri* NRRL 181]	1407	100%	89%
	11571	731	2	|EAL89589.1| lanosterol synthase, putative [*Aspergillus fumigatus* Af293]	1153	96%	76%
**Unknown AUPTS**	606	387	1	|EAA65430.1| hypothetical protein AN0654.2 [*Aspergillus nidulans* FGSC A4]	696	98%	86%
	7669	362	2	gb|EAA59165.1| hypothetical protein AN8143.2 [*Aspergillus nidulans* FGSC A4]	593	100%	83%
	11565	692	7	|EAA64518.1| hypothetical protein AN2407.2 [*Aspergillus nidulans* FGSC A4]	759	99%	53%
	13624	689	7	|EAU32979.1| conserved hypothetical protein [*Aspergillus terreus* NIH2624]	1036	100%	72%
	329	336	2	|CAK48316.1| unnamed protein product [*Aspergillus niger*]	529	99%	74%
	3446	456	1	|EAA63201.1| hypothetical protein AN2767.2 [*Aspergillus nidulans* FGSC A4]	832	98%	90%
	6064	404	3	|CCD54927.1| BcSTC5, similar to sesquiterpene cyclase [*Botryotinia fuckeliana*]	38.1	18%	31%
	6298	345	1	|EAA59634.1| hypothetical protein AN8012.2 [*Aspergillus nidulans* FGSC A4]	643	99%	88%

Note: AUPTS: *Aspergillus ustus* presumed terpene synthases; Prefix "Au" for all the ID of the AUPTS

Table S3 Primers used for gene deletion and complementation of *Au*3446

Type	Full name	Sequence: 5'—3' (restriction endonuclease)	Product size (bp)
pUC18	M13-47	CGCCAGGGTTTTCCCAGTCACGAC	156
	RV-M	GAGCGGATAACAATTTCACACAGG	
Selective marker	*Sh ble*-s	CCCACACACCATAGCTTCAAAAT	1172
	Sh ble-a	AGCTTGCAAATTAAAGCCTTCG	
	Hpt II-s	AAATTGACGCTTAGACAACTTAA	2146
	Hpt II -a	GCAGCTTGCCAACATGGTG	
*Au*3446 deletion	*Au*3446L′-s	CGGAATTCATCTTGACGGATTC (*Eco*RI)	1224
	*Au*3446L′-a	GGGGTACCTTGCGTGTTTGG (*Kpn*I)	1657
	*Au*3446R′-s	AACTGCAGCTAATCGAGATGTGC (*Pst*I)	
	*Au*3446R'-a	CCCAAGCTTCCTTCAAAATGG (*Hind*III)	
	*Au*3446-s	AAAGATGTTTCTCCCTTTGGCG	1167
	*Au*3446-a	GAGGTCGCGGGCCTGATA	
*Au*3446 complementation	*Au*3446L'-*Au*3446-*s*	GGGGTACCATACACTCTCATCCTAC (*Kpn*I)	2502
	*Au*3446L'-*Au_3446-a*	CTGCAGAACCAATGCATTGGCCGGTTCATGGCC (*Bst*XI)	
	Au_3446R'-s	CTGCAGAAAAAAAAGCTCGCTTTTGCAC (*Pst*I)	1012
	Au_3446R'-a	AAGCTTTGAACTTTTCTAGCCCGCCC (*Hind*III)	

Table S4 Plasmids used for gene deletion and complementation of *Au*3446

Type	Serial number / Full name	Characteristics
Expression vectors	pGAPZαA	P_{GAP}, α-factor signal, myc epitope tag, C-terminal polyhistidine tag, T_{Aox1}, P_{Tef1}, P_{Em7}, *Sh ble* (Zeocin^r), T_{Cyc1}, pUC ori (ColE1 ori)
Cloning vectors	pMD18-T	Derived from pUC18, pUC ori (ColE1 ori), O_{LacZ}, *bla* (Amp^r)
	pMD18-T-Simple	Derived from pMD18-T, without multiple cloning site
	pCAMBIA1301	$P_{CAMV35S}$, *Gus*, $T_{Nos\ polyA}$, $P_{CAMV35S}$, *HptII* (HmB^r), $T_{CAMV35S\ polyA}$, pVS1 rep, pVS1 sta, pBR322 ori, pBR322 born, Kan^r
	pTFCM	Derived from pCAMBIA1301, $P_{CAMV35S}$ and *HptII* (HmB^r) were substituted with P_{TrpC}, *HptII* (HmB^r), and T_{TrpC}
Random insertion vectors	JS	P_{TrpC}, *eGFP*, T_{TrpC}, *HptII* (HmB^r), pVS1 rep, pVS1 sta, Kan^r
Deletion and complementation vectors	pWHU2201 / pUC18 / P_{Tef1}-P_{Em7}-*Sh ble*-T_{Cyc1}	Derived from pMD18-T and pGAPZαA, pUC ori (ColE1 ori), O_{LacZ}, *bla* (Amp^r), P_{Tef1}, P_{Em7}, *Sh ble* (Zeocin^r), T_{Cyc1}
	pWHU2202 / pUC18 / $P_{CAMV35S}$-*HptII*-$T_{CAMV35S\ polyA}$	Derived from pMD18-T and pCAMBIA1301, pUC ori (ColE1 ori), O_{LacZ}, *bla* (Amp^r), $P_{CAMV35S}$, *HptII* (HmB^r), $T_{CAMV35S\ polyA}$
Genetic deletion and complementation system (gene *Au_3446*)	pUC18 / *Au_3446L*	Derived from pMD18-T-Simple
	pUC18 / *Au_3446R*	Derived from pMD18-T-Simple
	pUC18 / *Au_3446L'*	Derived from pMD18-T-Simple
	pUC18 / *Au_3446R'*	Derived from pMD18-T-Simple
	pUC18 / *Au_3446L'*-P_{Tef1}-P_{Em7}-*Sh ble*-T_{Cyc1}	Derived from pUC18 / P_{Tef1}-P_{Em7}-*Sh ble*-T_{Cyc1}
	pWHU2203 / pUC18 / *Au_3446L'*-P_{Tef1}-P_{Em7}-*Sh ble*-T_{Cyc1}-*Au_3446R'*	Derived from pUC18 / *Au_3446L'*-P_{Tef1}-P_{Em7}-*Sh ble*-T_{Cyc1}
	pWHU2204 / JS / *Au_3446L'*-P_{Tef1}-P_{Em7}-*Sh ble*-T_{Cyc1}-*Au_3446R'*	Derived from JS and pUC18 / *Au_3446L'*-P_{Tef1}-P_{Em7}-*Sh ble*-T_{Cyc1}-*Au_3446R'*, P_{TrpC}, *eGFP*, and T_{TrpC} were substituted with *Au_3446L'*-P_{Tef1}-P_{Em7}-*Sh ble*-T_{Cyc1}-*Au_3446R'*
	pUC18 / *Au_3446*-*Au_3446L'*-$P_{CAMV35S}$-*HptII*- $T_{CAMV35S\ polyA}$	Derive from pUC18 / $P_{CAMV35S}$-*HptII*-$T_{CAMV35S\ polyA}$
	pWHU2205 / pUC18 / *Au_3446*-*Au_3446L'*-$P_{CAMV35S}$-*HptII*- $T_{CAMV35S\ polyA}$-*Au_3446R'*	Derive from pUC18 / *Au_3446*-*Au_3446L'* - $P_{CAMV35S}$-*HptII*-$T_{CAMV35S\ polyA}$

Table S5 Primers used for gene cluster inactivation

Type	Full name	Sequence: 5'—3' (restriction endonuclease)	Product size (bp)
pUC18	M13-47	CGCCAGGGTTTTCCCAGTCACGAC	156
	RV-M	GAGCGGATAACAATTTCACACAGG	
Selective marker	*Sh ble*-s	CCCACACACCATAGCTTCAAAAT	1172
	Sh ble-a	AGCTTGCAAATTAAAGCCTTCG	
	HptII-s	AAATTGACGCTTAGACAACTTAA	2146
	HptII-a	GCAGCTTGCCAACATGGTG	
POC*3446* deletion	POC*3446L*-s	GAATTCCCACAGATAGTATGCGAGTC (*Eco*RI)	1419
	POC*3446L*-a	TCTAGACAAACGCCAGAACATCACG (*Xba*I)	
	POC*3446R*-s	GTCGACTAGGGTGGTATTTGCGT (*Sal*I)	1128
	POC*3446R*-a	AAGCTTGGGCATCCGTATGATGTCG (*Hind*III)	
POC*8003* deletion	POC*8003L*-s	GAATTCGTCGTACAGTCAGGTAGGGA (*Eco*RI)	1085
	POC*8003L*-a	GGTACCAAGGAGGAGTGCAAGGAA (*Kpn*I)	
	POC*8003R*-s	CCTGCAGGTTGCCGAGGACAAGG (*Sbf*I)	1061
	POC*8003R*-a	AAGCTTCATCTGTGGACGGAGTAAAG (*Hind*III)	
	Au_8003-s	CGCCCACCACTCATTCTTTTAG	1063
	Au_8003-a	CGACCTGATCCGATGCTGTC	
POC*13192* deletion	POC*13192L*-s	GAATTCTAACTTCAAATCAGCGAGGA (*Eco*RI)	1224
	POC*13192L*-a	TCTAGAATACCCACGGACCCAAC (*Xba*I)	
	POC*13192R*-s	CCTGCAGGAGCAGCATCTTCGATTGG (*Sbf*I)	1077
	POC*13192R*-a	AAGCTTATTCTGGGACAGGAACTAG (*Hind*III)	
	Au_13192-s	AAGAACAAGCACGGACTGCC	1096
	Au_13192-a	CCTGCCTCGTCCAATACAGAT	
POC*11565* deletion	POC*11565L*-s	GAATTCTTGTTGAGGCGTTTTGTG (*Eco*RI)	1058
	POC*11565L*-a	TCTAGAGTCATCGTGCGAGTAGG (*Xba*I)	
	POC*11565R*-s	CCTGCAGGCATCCGCAGTCATAGTAGT (*Sbf*I)	1018
	POC*11565R*-a	AAGCTTCGCCTCCACGTTG (*Hind*III)	
	Au_11565-s	CTCGTATTCGCTTTGGGTTGA	1106
	Au_11565-a	CCAGCATTGTCGGGTTCG	
POC*6298* deletion	*POC6298L*-s	GAATTCGCAGCCAGTTCCTTCCCTA (*Eco*RI)	1508
	POC6298L-a	GGATCCTTTTTACCCAAGCCCATC (*Bam*HI)	
	POC6298R-s	CCTGCAGGAGGGAAATACCGCCTCAAAAG (*Sbf*I)	1208
	POC6298R-a	AAGCTTAGCCTCCTCCTCGTTC (*Hind*III)	
	Au_6298-s	TTTGAGGCGGTATTTCCC	1038
	Au_6298-a	ACCTCCTTCTTCAGTCCCT	

Table S6 Plasmids used for gene cluster inactivation

Type	Serial number / Full name	Characteristics
Expression vectors	pGAPZαA	P_{GAP}, α-factor signal, myc epitope tag, C-terminal polyhistidine tag, T_{Aox1}, P_{Tef1}, P_{Em7}, *Sh ble* (Zeocin^r), T_{Cyc1}, pUC ori (ColE1 ori)
Cloning vectors	pMD18-T	Derived from pUC18, pUC ori (ColE1 ori), O_{LacZ}, *bla* (Amp^r)
	pMD18-T-Simple	Derived from pMD18-T, without multiple cloning site
	pCAMBIA1301 (CAMBIA, Canberra, Australia)	$P_{CAMV35S}$, *Gus*, $T_{Nos\ polyA}$, $P_{CAMV35S}$, *HptII* (HmB^r), $T_{CAMV35S\ polyA}$, pVS1 rep, pVS1 sta, pBR322 ori, pBR322 born, Kan^r
	pTFCM	Derived from pCAMBIA1301, $P_{CAMV35S}$ and *HptII* (HmB^r) were substituted with P_{TrpC}, *HptII* (HmB^r), and T_{TrpC}
POC*3446* deletion	pUC18 / POC*3446L*	Derived from pMD18-T-Simple
	pUC18 / POC*3446R*	Derived from pMD18-T-Simple
	pUC18 / POC*3446L*-P_{Tef1}-P_{Em7}-*Sh ble*-T_{Cyc1}	Derived from pUC18 / P_{Tef1}-P_{Em7}-*Sh ble*-T_{Cyc1}
	pWHU2206 / pUC18 / POC*3446L*-P_{Tef1}-P_{Em7}-*Sh ble*-T_{Cyc1}-POC*3446R*	Derived from pUC18 / POC*3446L*-P_{Tef1}-P_{Em7}-*Sh ble*-T_{Cyc1}
	pWHU2207 / JS / POC*3446L*-P_{Tef1}-P_{Em7}-*Sh ble*-T_{Cyc1}-POC*3446R*	Derived from JS and pUC18 / POC*3446L*-P_{Tef1}-P_{Em7}-*Sh ble*-T_{Cyc1}-POC*3446R*, P_{TrpC}, *eGFP*, and T_{TrpC} were substituted with POC*3446L*-P_{Tef1}-P_{Em7}-*Sh ble*-T_{Cyc1}-POC*3446R*
POC*8003* deletion	pUC18 / *POC8003L*	Derived from pMD18-T-Simple
	pUC18 / *POC8003R*	Derived from pMD18-T-Simple
	pUC18 / POC*8003L*-P_{Tef1}-P_{Em7}-*Sh ble*-T_{Cyc1}	Derived from pUC18 / P_{Tef1}-P_{Em7}-*Sh ble*-T_{Cyc1}
	pWHU2212 / pUC18 / POC*8003L*-P_{Tef1}-P_{Em7}-*Sh ble*-T_{Cyc1}-POC*8003R*	Derived from pUC18 / POC*8003L*-P_{Tef1}-P_{Em7}-*Sh ble*-T_{Cyc1}
POC*13192* deletion	pUC18 / POC*13192L*	Derived from pMD18-T-Simple
	pUC18 / POC*13192R*	Derived from pMD18-T-Simple
	pUC18 / POC*13192L*-P_{Tef1}-P_{Em7}-*Sh ble*-T_{Cyc1}	Derived from pUC18 / P_{Tef1}-P_{Em7}-*Sh ble*-T_{Cyc1}
	pWHU2213 / pUC18 / POC*13192L*-P_{Tef1}-P_{Em7}-*Sh ble*-T_{Cyc1}-POC*13192R*	Derived from pUC18 / POC*13192L*-P_{Tef1}-P_{Em7}-*Sh ble*-T_{Cyc1}
POC*11565* deletion	pUC18 / POC*11565L*	Derived from pMD18-T-Simple
	pUC18 / POC*11565R*	Derived from pMD18-T-Simple
	pUC18 / POC*11565L*-P_{Tef1}-P_{Em7}-*Sh ble*-T_{Cyc1}	Derived from pUC18 / P_{Tef1}-P_{Em7}-*Sh ble*-T_{Cyc1}
	pWHU2214 / pUC18 / POC*11565L*-P_{Tef1}-P_{Em7}-*Sh ble*-T_{Cyc1}-POC*11565R*	Derived from pUC18 / POC*11565L*-P_{Tef1}-P_{Em7}-*Sh ble*-T_{Cyc1}
POC*6298* deletion	pUC18 / POC*6298L*	Derive from pMD18-T-Simple
	pUC18 / POC*6298R*	Derive from pMD18-T-Simple
	pUC18 / POC*6298L*-P_{Tef1}-P_{Em7}-*Sh ble*-T_{Cyc1}	Derived from pUC18 / P_{Tef1}-P_{Em7}-*Sh ble*-T_{Cyc1}
	pWHU2223 / pUC18 / POC*6298L*-P_{Tef1}-P_{Em7}-*Sh ble*-T_{Cyc1}-POC*6298R*	Derived from pUC18 / POC*6298L*-P_{Tef1}-P_{Em7}-*Sh ble*-T_{Cyc1}

Table S7 BlastnP result in GenBank database of 5 TS/TC proteins with reference sequences. Underlined sequences were added in 2015; shaded were unidentified sequences

	sequence	sources	description		
			Max score	Query cover	Ident
Au8003	AcOS	*Aspergillus clavatus*	979	100%	65%
	EvVS	*Emericella variecolor*	273	85%	45%
	EvSS	*Emericella variecolor*	552	100%	41%
	PaPS	*Phomopsis amygdali*	516	100%	40%
	PaFS	*Phomopsis amygdali*	517	100%	39%
	AbFS	*Alternaria brassicicola*	466	98%	36%
	NfSS	*Neosartorya fischeri*	290	96%	30%
Au13192	EvVS	*Emericella variecolor*	1115	99%	78%
	NfSS	*Neosartorya fischeri*	218	83%	46%
	PaFS	*Phomopsis amygdali*	425	98%	36%
	PaPS	*Phomopsis amygdali*	394	97%	35%
	EvSS	*Emericella variecolor*	412	98%	34%
	AbFS	*Alternaria brassicicola*	359	96%	34%
	AcOS	*Aspergillus clavatus*	402	98%	33%
Au11565	NfSS	*Neosartorya fischeri*	191	87%	40%
	AcOS	*Aspergillus clavatus*	263	96%	32%
	EvSS	*Emericella variecolor*	274	96%	30%
	EvVS	*Emericella variecolor*	237	75%	29%
	PaFS	*Phomopsis amygdali*	209	73%	29%
	AbFS	*Alternaria brassicicola*	206	71%	28%
	PaPS	*Phomopsis amygdali*	259	98%	28%
Au6298	WcFPPS	*Wolfiporia cocos*	397	98%	58%
	CpFPPS	*Chimonanthus praecox*	326	100%	50%
	EpFPPS	*Euphorbia pekinensis*	318	100%	49%
	OsaFPPS	*Ornithogalum longebracteatum*	315	98%	48%
	RpFPPS1	*Ornithogalum longebracteatum*	310	97%	48%
	RpFPPS2	*Ornithogalum longebracteatum*	315	97%	47%
	RsFPPS	*Rhizosolenia setigera*	319	99%	46%
Au3446	Nf	*Neosartorya fischeri*	786	100%	86%
	Ao	*Aspergillus oryzae*	776	100%	85%
	An	*Aspergillus niger*	745	100%	79%
	Ak	*Aspergillus kawachii*	742	100%	79%
	Ml	*Micrococcus luteus*	125	60%	32%
	Ss	*Sulfolobus solfataricus*	49.3%	34%	26%

Table S8 Primers used for gene expression

Type	Full name	Sequence: 5'—3' (restriction endonuclease)	Product size (bp)
*Au*8003 expression	*Au*8003-F	CATATGATGGAGTATAAGTACTCGACC(*Nde* I)	2178
	*Au*8003-R	AAGCTT TCAAACCTTCAGCAGCTCCA(*Hind* III)	
*Au*6298 expression	*Au*6298-F	CCCATATGATGTCTTCACCGCGTGCC（*Nde* I）	1038
	*Au*6298-R	CCAAGCTTTTATTTGGTGCGCTTGTAAA（*Hind* III）	
*Au*11565 expression	*Au*11565	GGGGTACCGCGATGTACACCCTCGA（*Kpn* I）	2405
	*Au*11565	GCTCTAGATACACTTTCAGCAAAAGCA（*Xba* I）	
Au13192 exon fusion and expression	*Au*13192E1-s	AAGCTTATGCCACAAA CACAATCC(*Hind*III)	326
	*Au*13192E1-a	GCCGACTCGACAACATTATCATACAGAAAC GCATACTCAAAGATATAACAAATAA	
	*Au*13192E3-s	TGATAATGTTGTCGAGTCGGCGGCCAATTC TACGTTGAACATGGACACGGACAA	301
	*Au*13192E3-a	CATGAGCATGTCGACGAAGGGGGCGCCGGTATCGAT	
	*Au*13192E4-s	CTTCGTCGACATGCTCATG	766
	*Au*13192E4-a	GCCGGGGCGAGGAGGATCTCGTCTCCTAGCTGCAC	
	*Au*13192E5-s	ATCCTCCTCGCCCCGGC	175
	*Au*13192E5-a	AGTCTTCGATGTCGTCGAGCATGAGCGAGGCATTGTGTA	
	*Au*13192E6-s	CTCGACGACATCGAAGACT	309
	*Au*13192E6-a	CTGCACCATCAACCTCGTAAGCAACCGGAAC AACCCACCGGTCTTCTGCCTAACCATCTCCAAAT	
	*Au*13192E8-s	TTACGAGGTTGATGGTGCAGATTGCGCCGGT GCGGCGGAAAGATCTCGACGGCATCTTATCAT	126
	*Au*13192E8-a	CCCTTTTGGCCGGTGTACTCTTCAGTTAGGTTCTTGTAGT	
	*Au*13192E9-s	AGTACACCGGCCAAAAGGG	333
	*Au*13192E9-a	GCGGCCGCTACACCTTCAACCTCTGCAC(*Not*I)	

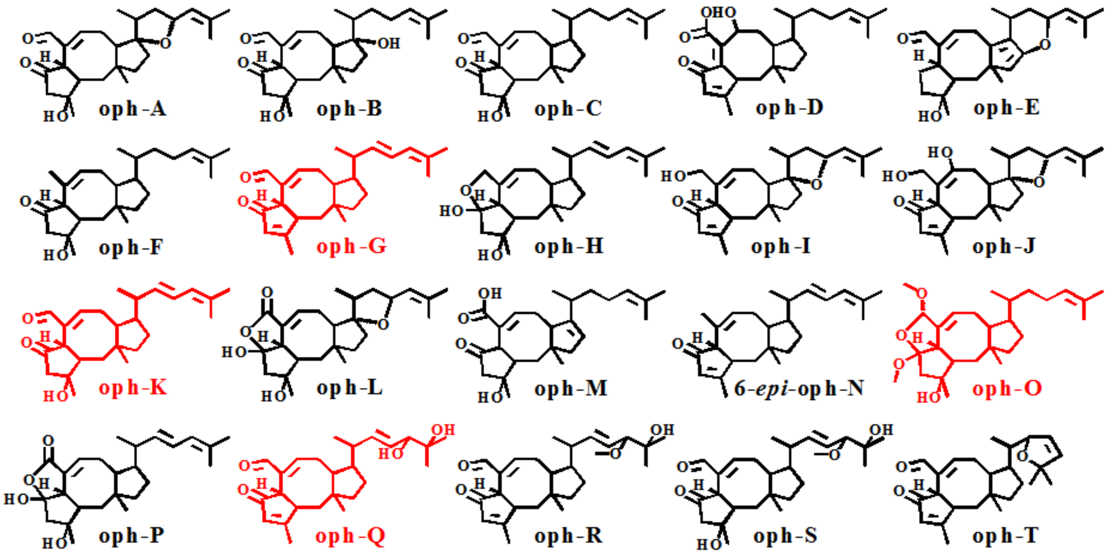

Figure S1 Twenty reported subgroups (1957–2013) of ophiobolins with 5-8-5 ring scaffolds. These structures were from references 12,14,15 and 16; red color shows ophiobolins identified from *Aspergillus ustus* 094102

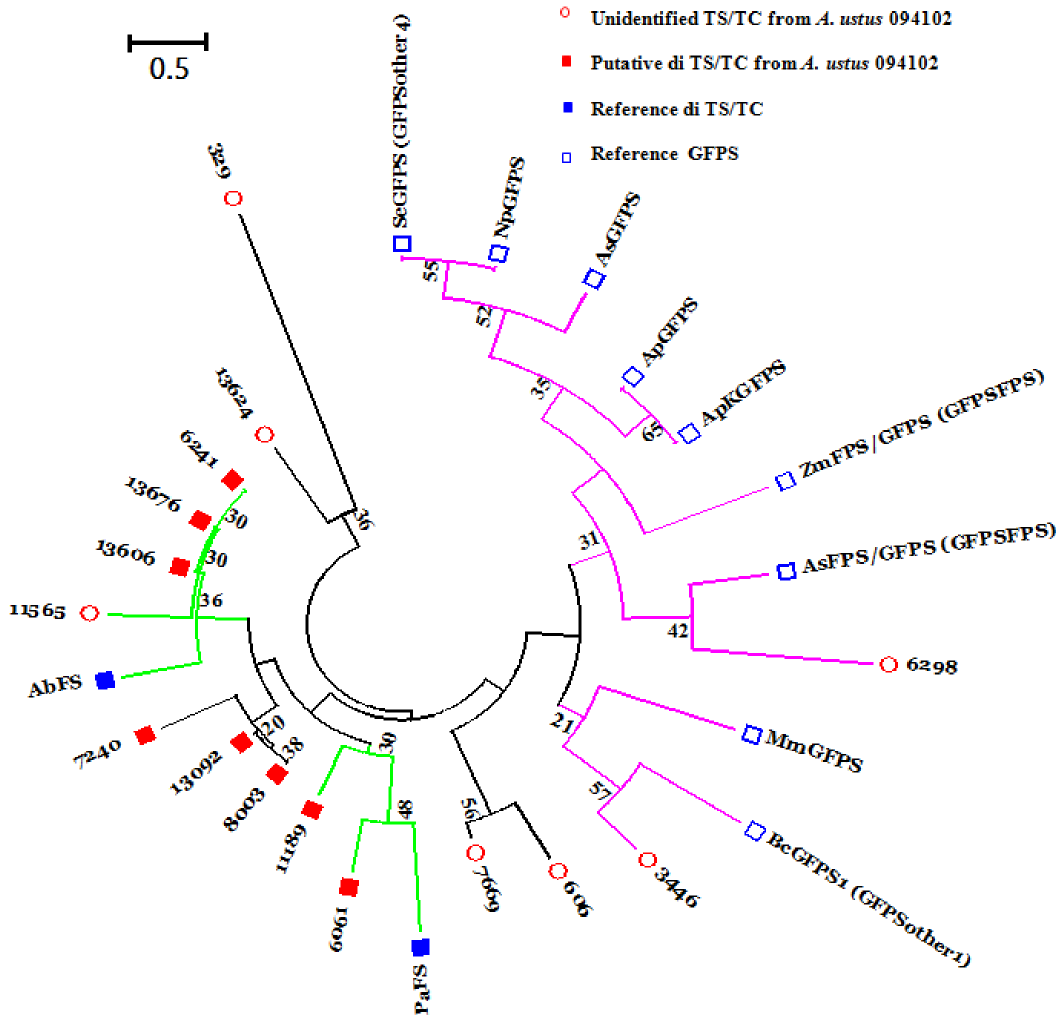

Figure S2 Neighbour-joining phylogenetic tree based on amino acid sequences of chain length determination motif (Hisashi et al[25]) of the 15 trans-IPP terpene synthetase proteins. The tree was generated based on Kimura 2-parameter matrix in MEGA software (version 5.0). Numbers at nodes indicate bootstrap values with 1000 iterations. Bar, 0.5, substitutions per nucleotide position. PaPS (*Phomopsis amygdali* phomopsene synthase); PaFS (*Phomopsis amygdali* fusicoccadiene synthase); NpGFPPS (GFPPS from *Natronobacterium pharaonis*); ApGFPPS (GFPPS from *Aeropyrum pernix*); MmGFPPS (GFPPS from *Methanosarcina mazei*)

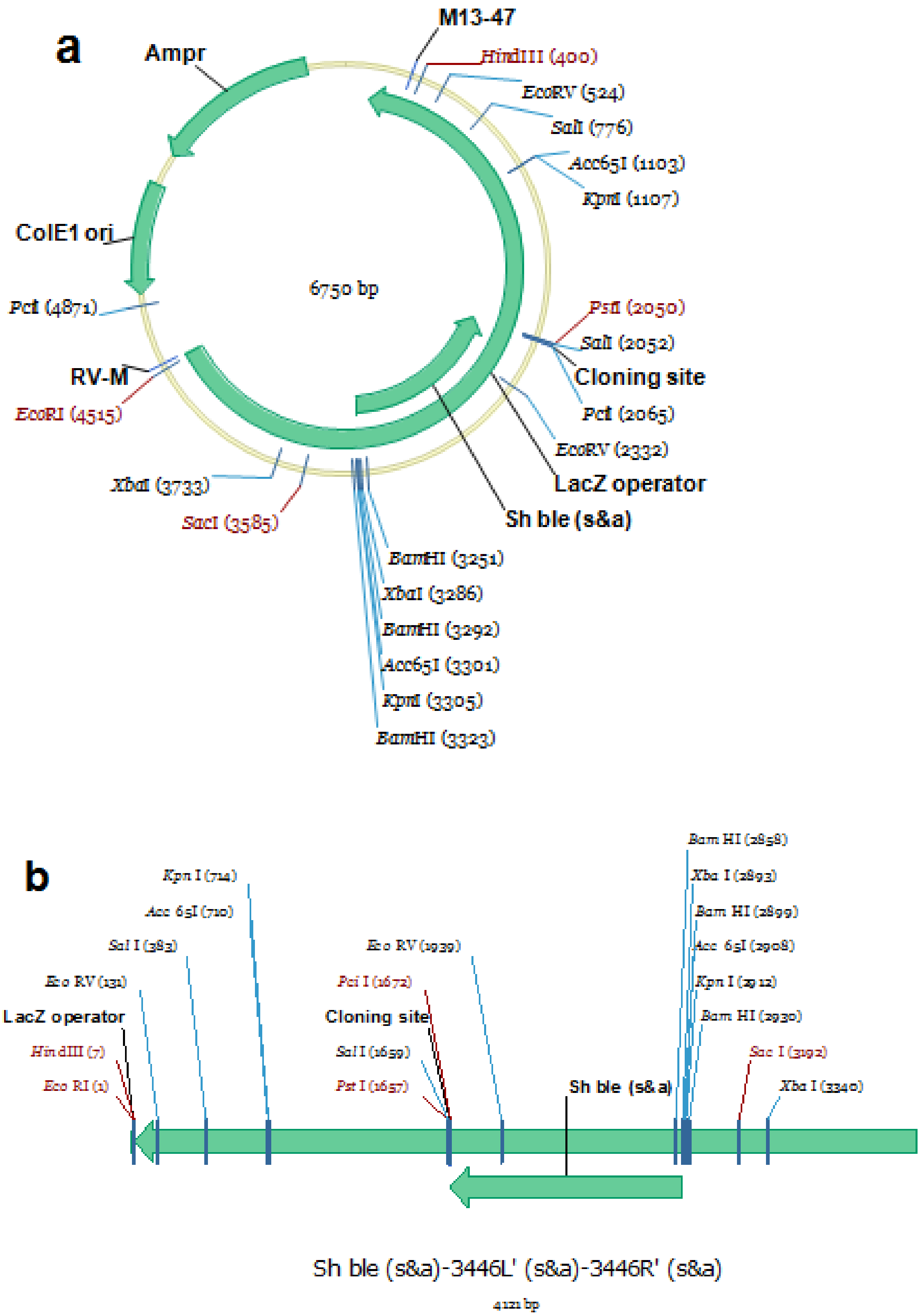
a
Ampr
M13-47
HindIII (400)
EcoRV (524)
SalI (776)
Acc65I (1103)
KpnI (1107)
ColE1 ori
6750 bp
PciI (4871)
PstI (2050)
SalI (2052)
Cloning site
PciI (2065)
RV-M
EcoRI (4515)
EcoRV (2332)
LacZ operator
Sh ble (s&a)
XbaI (3733)
SacI (3585)
BamHI (3251)
XbaI (3286)
BamHI (3292)
Acc65I (3301)
KpnI (3305)
BamHI (3323)
b
Kpn I (714)
Acc 65I (710)
Sal I (383)
Eco RV (131)
LacZ operator
Hin dIII (7)
Eco RI (1)
Eco RV (1939)
Pci I (1672)
Cloning site
Sal I (1659)
Pst I (1657)
Sh ble (s&a)
Bam HI (2858)
Xba I (2893)
Bam HI (2899)
Acc 65I (2908)
Kpn I (2912)
Bam HI (2930)
Sac I (3192)
Xba I (3340)
Sh ble (s&a)-3446L' (s&a)-3446R' (s&a)
4121 bp

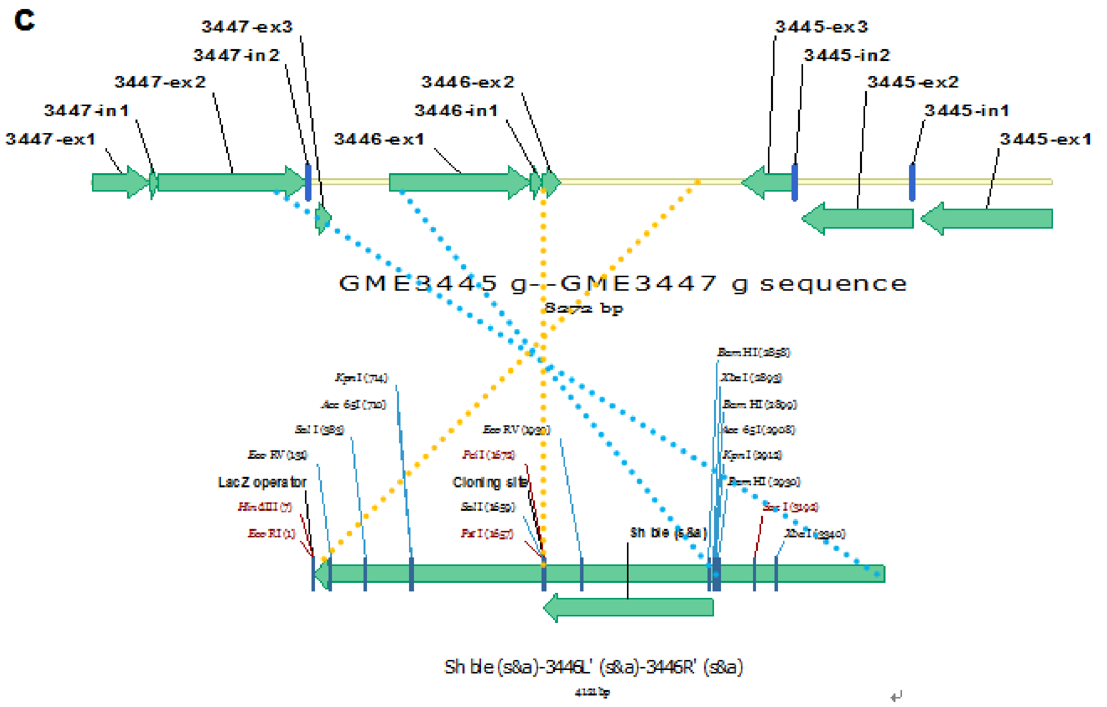

Figure S3 Gene deletion of Au3446. (a) Plasmid pMD18-T-3446L'-Sh ble- 3446R'constructed with the selective marker and the left and right homologous sequences for recombination; (b) The detailed targeting fragment; (c) Homologous recombinant of the cassette with the genomic DNA to delete the target gene.

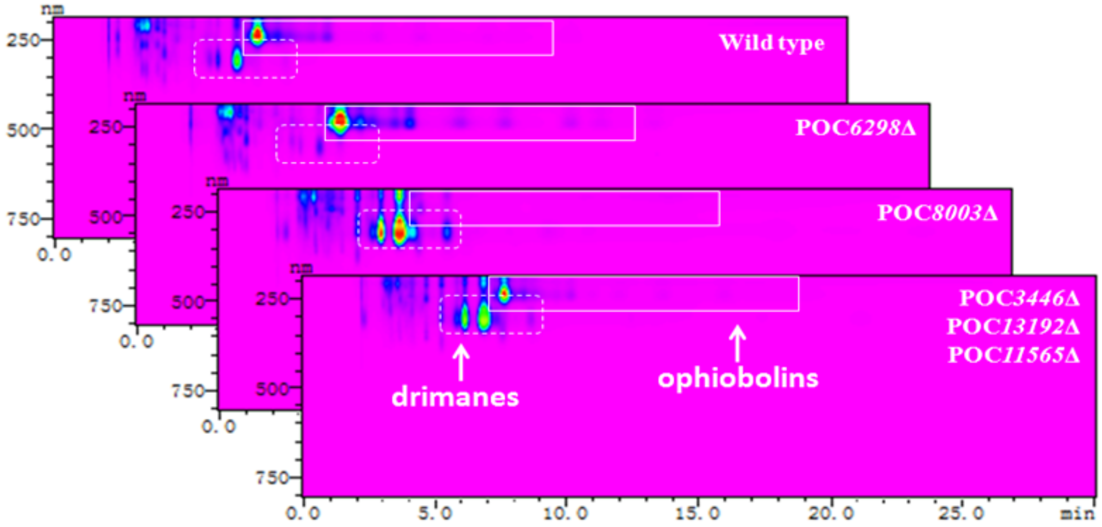

Figure S4 Full wavelength range from 210–400 nm analysis collected for ophiobolin and drimane on the fermentation products of wild type strain and the five gene cluster inactivated mutants. Ophiobolin did not appear for ΔPOC8003, increased for ΔPOC6298, partially decreased for ΔPOC13192, ΔPOC3446 and ΔPOC11565; drimane was increased for ΔPOC8003, ΔPOC13192, ΔPOC3446 and ΔPOC11565, but decreased for ΔPOC6298.

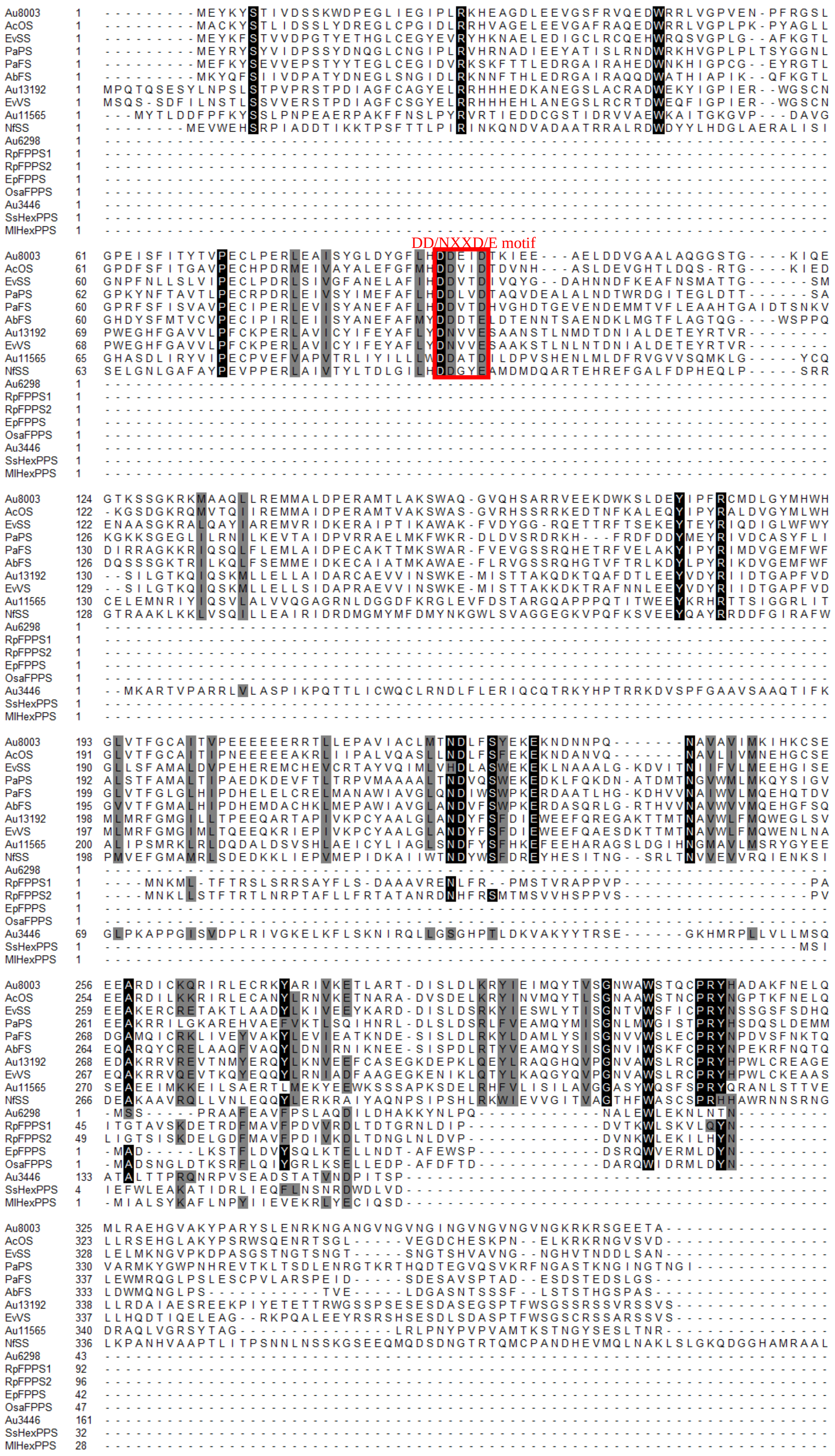
DD/NXXD/E motif

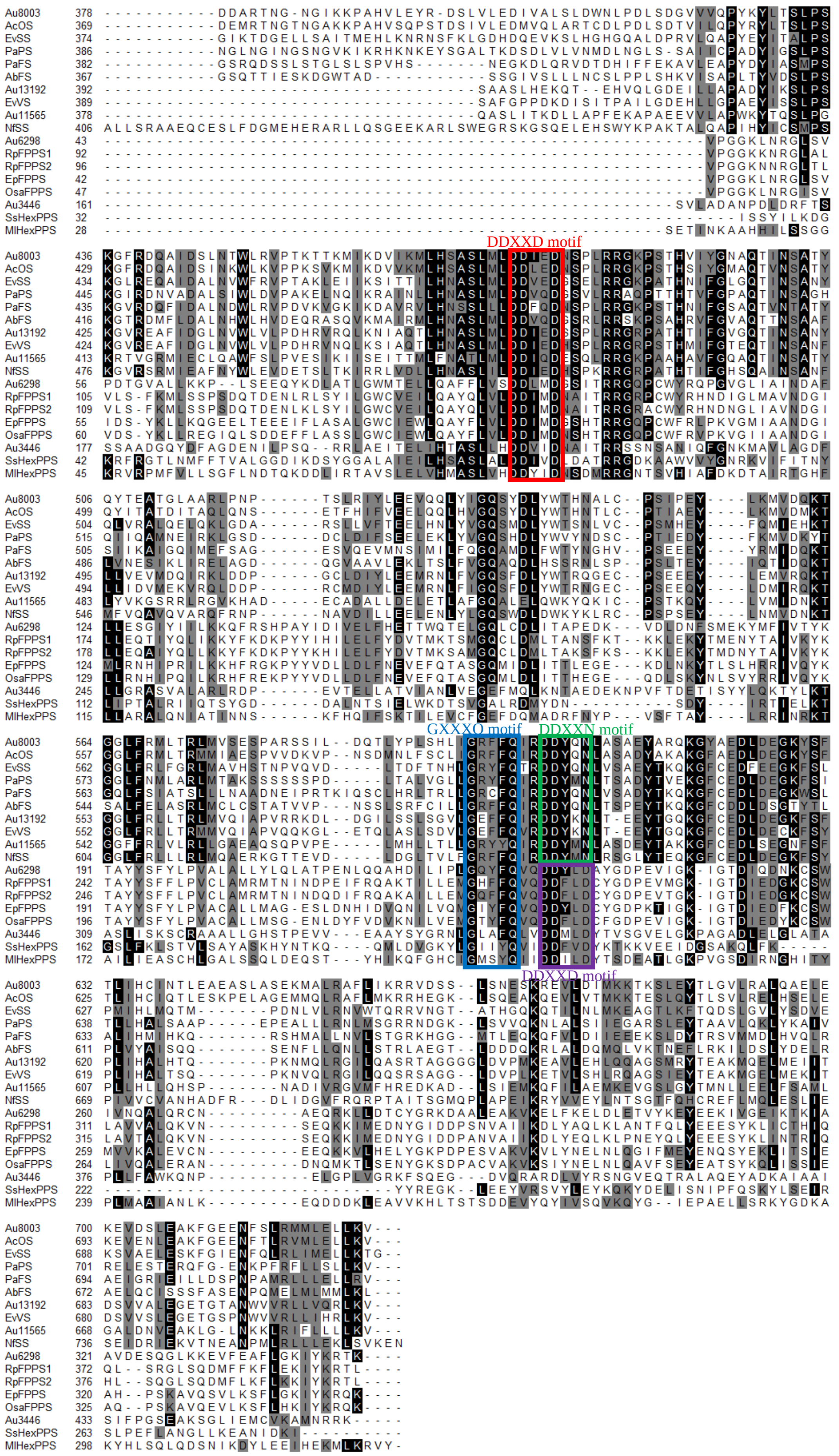
DDXXD motif
GXXXQ motif
DDXXN motif
DDXXD motif

Figure S5. Alignment of amino acid sequences of the five terpene synthesis related proteins in ophiobolin biosynthesis with identified functions. Shown here are DDXXD/E (Terpene cyclase (TC) domain), DDXXD (Prenyltransferases (PT) domain), GQ(PT), DDXXN (bifunction PT only) DDXXD (single function PT only), *Aspergillus clavatus* ophiobolin F synthase (AcOS A1C8C3), Emericella variecolor stellata-2,6,19-triene Synthase (EvSS LC073704), *Phomopsis amygdali* phomopsene synthase (PaPS AB254159), *Phomopsis amygdali* fusicoccadiene synthase (PaFS AB267396), *Alternaria brassicicola* fusicoccadiene synthase (AbFS C9K2Q3), *Emericella variecolor* Variediene Synthase (EvVS LC063849), *Neosartorya fischeri* sesterfisherol synthase (NfSS EAW16201), *Rhopalosiphum padi* isoprenyl diphosphate synthase (RpFPPS1 and RpFPPS2 HQ850372 and HQ850373), *Ornithogalum saundersiae* farnesyl pyrophosphate synthase (OsaFPPS KF509889), *Euphorbia pekinensis* farnesyl diphosphate synthase (EpFPPS FJ755465), *Sulfolobus solfataricus* Hexaprenyl Pyrophosphate Synthase (SsHexPPS 2AZK_B), and *Micrococcus luteus* heterodimeric hexaprenyl diphosphate synthase (MlHexPPS 3AQB_B).

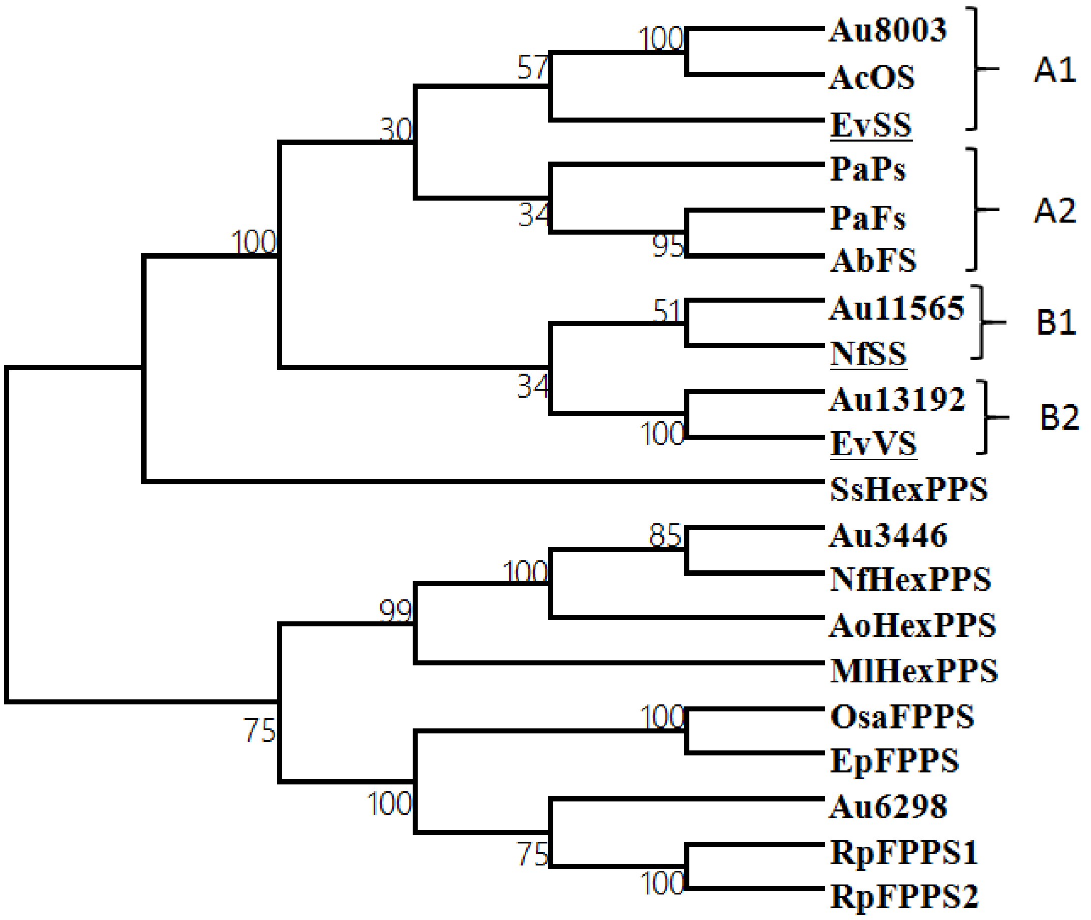

Figure S6 Neighbour-joining phylogenetic tree based on amino acid sequences of bifunctional terpene synthases, farnesyl pyrophosphate synthases and hexprenyl pyrophosphate synthases. The tree was generated based on Kimura 2-parameter matrix in MEGA software (version 6.0). Numbers at nodes are bootstrap values obtained using 1000 iterations. The scale bar indicates 0.2 substitutions per nucleotide position. Reference sequences were the same as in Figure S5.

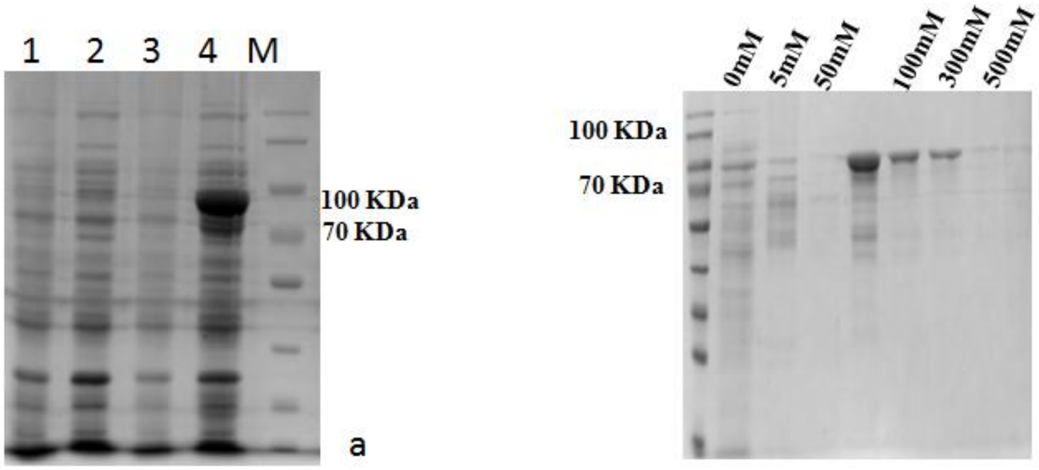

Figure S7. SDS-PAGE analysis of expression and purification of Au8003. (a) Overexpression of Au8003 gene in BL21(DE3). Lane M: Marker; Lane 1: uninduced (without isopropyl (3-D-thiogalactopyranoside) IPTG) bacterial protein carrying plasmid pET28a; Lane 2: induced (with IPTG) bacterial protein carrying plasmid pET28a, Lane 3: uninduced bacterial protein carrying the Au8003 cDNA; Lane 4: induced bacterial protein carrying the Au8003 cDNA; (b) purification with different concentrations of imidazole.

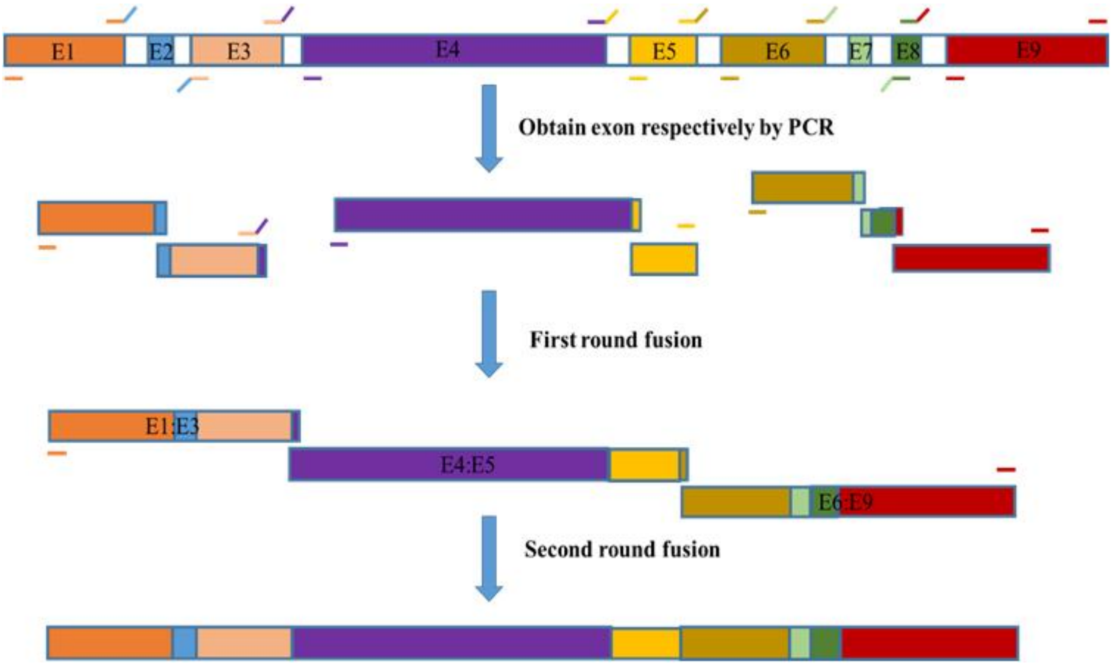

Figure S8. Strategy of Au13192 multiple exon fusion by overlap extension PCR. Stage 1: designing primers and amplifying exon 1 (E1) to exon 9 (E9) with overlap sequences through normal PCR (because the E2 and E7 segments are too short, the two gene sequences were designed according to the adjacent two exons of the reverse primer and forward primer, respectively); Stage 2: first round fusion obtained E1:E3/E4:E5/E6:E9, using adjacent exon as template with forward primers of front end exon and reverse primers of tail end exon; Stage3: second fusion obtained Au13192 cDNA following stage 2.

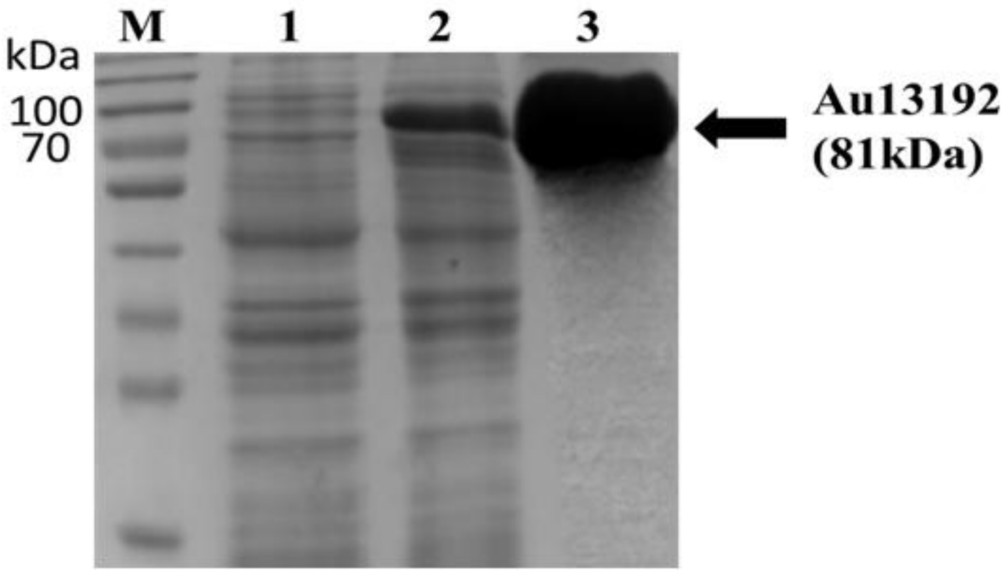

Figure S9. SDS page gel detection of overexpression and purification of Au13192. Lane M: Marker; Lane 1: uninduced (without isopropyl (3-D-thiogalactopyranoside) bacterial protein carrying the 2.1-kb Au13192 cDNA; Lane 2: induced (with isopropyl p-D-thiogalactopyranoside) bacterial protein carrying the 2.1-kb Au13192 cDNA; Lane3: purified Au13192

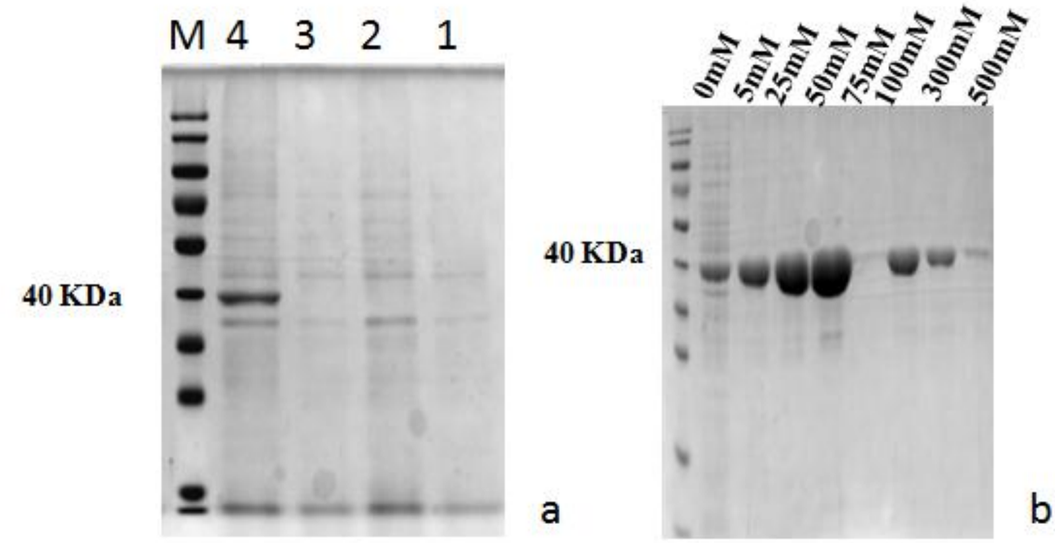

Figure S10. SDS-PAGE analysis of expression and purification of Au 6298. (a) Overexpression of Au6298 genes in BL21(DE3). Lane M: Marker; Lane 1: uninduced (without isopropyl (3-D-thiogalactopyranoside) IPTG) bacterial protein carrying plasmid pET28a; Lane 2: induced (with IPTG) bacterial protein carrying plasmid pET28a, Lane 3: uninduced bacterial protein carrying the Au6298 cDNA; Lane 4: induced bacterial protein carrying the Au6298 cDNA; (b) purification with different concentrations of imidazole.

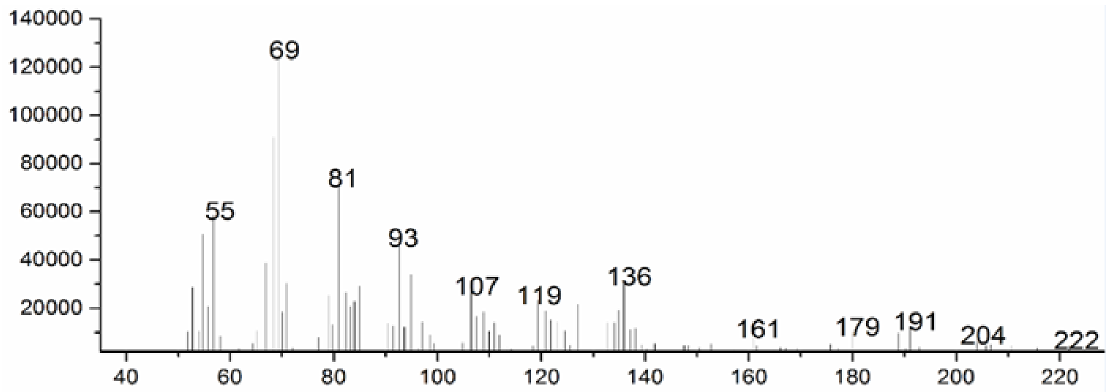

Figure S11 Mass spectrum of authentic farnesol.
